# A workflow to explore elongase diversity and extend the repertoire of fatty acids produced by *Yarrowia lipolytica*

**DOI:** 10.1186/s12934-025-02890-y

**Published:** 2025-12-23

**Authors:** Jérémy Le Reun, Zélie Salvioli, Christian Croux, Jérémy Esque, Isabelle André, Florence Bordes

**Affiliations:** Toulouse Biotechnology Institute, TBI, Université de Toulouse, CNRS, INRAE, INSA, Toulouse, France

**Keywords:** *Yarrowia lipolytica*, Fatty acids, Very-long chain fatty acids, Fatty acid elongases, Enzyme characterization, Substrate specificity, Miniaturization

## Abstract

**Background:**

Fatty acids display highly diverse structures that confer these molecules unique chemical properties and distinct physiological functions. Identifying the substrate specificity of enzymes active on fatty acids is crucial, both for understanding their function in natural organisms and for developing efficient cell factories to produce original fatty acids. However, these enzymes are often membrane-bound and/or act on esterified substrates and studying them in vitro is thus challenging. This is why in vivo characterization of these enzymes’ specificity is an interesting approach. Herein, we harness the industrially relevant oleaginous *Yarrowia lipolytica* as a chassis for characterizing heterologous enzymes active on fatty acids, which can be used to diversify its fatty acid composition. As a case study, we investigated fatty acid elongases (ELO) responsible for the synthesis of very long-chain fatty acids (> 20 carbons), which are specific of given chain lengths and/or unsaturation patterns. Despite their interest, investigation and utilization of these membrane enzymes remain largely underexplored.

**Results:**

We developed a workflow for characterizing heterologous elongases in *Y. lipolytica*, addressing several limitations to increase throughput. First, we set up a strain engineering strategy to easily integrate the ELO cassettes into targeted loci using CRISPR-Cas9, where screening of homologous recombination events is facilitated by fluorescence. We demonstrated that the native elongase YlELO2, responsible for the elongation of saturated and monounsaturated fatty acids up to 26 carbons, has to be inactivated to avoid functional redundancy and finely characterize heterologous elongase specificity. As it is an essential gene, we designed an optimized strategy for YlELO2 Knock-Out by a Knock-In of the elongase cassette. We then miniaturized cultures and fatty acid extraction in 96-well plates format. Using this workflow, we characterized seven human elongases on endogenous fatty acids and on five exogenous polyunsaturated fatty acids in a single series of experiments.

**Conclusion:**

We have developed tools and methods to characterize elongase specificity, from strain design to fatty acid production and analysis. Applicable to any fatty acid–modifying enzymes, these methodological developments will be useful to expand the repertoire of enzymes usable in *Y. lipolytica* and pave the way to produce new original fatty acids in this chassis.

**Supplementary Information:**

The online version contains supplementary material available at 10.1186/s12934-025-02890-y.

## Background

Fatty acids (FAs) are essential building blocks for cellular physiology, involved in membrane structure, energy storage, and signaling. Their structures are highly diverse, with variations in chain length, degree of saturation, and functional group modifications, that confers these molecules unique chemical properties of interest for a variety of applications as biofuels, lubricants, and specialty chemicals [1–3]. Thus, elucidating fatty acid biosynthesis pathways and the enzymes involved is of utmost interest not only to gain fundamental knowledge but also to produce biosourced added-value molecules. Among original fatty acids, very-long chain fatty acids (VLCFAs), with chain lengths of 20 carbons and more, are of particular interest as they are involved in various major pathways and physiological functions. In plants, VLCFAs are required for the synthesis of cuticular waxes, pollen coat and suberin [4], while in mammals they have numerous functions in skin barrier formation, supporting retinal functions, resolving inflammation, sustaining myelin integrity, facilitating sperm development and maturation, and maintaining liver homeostasis [5]. VLCFAs and their derivatives are also valuable chemicals to produce lubricants, polymers, pharmaceuticals and cosmetics. Due to their low content in living organisms, natural sources of VLCFAs such as vegetable oils are limited in number and yield. To overcome this limitation, microbial production of VLCFAs has gained interest over the years as metabolic engineering can be easily used to finely tune fatty acid composition towards desired VLCFAs [6, 7]. Such approaches involve a controlled modulation of the fatty acid elongation pathway to achieve both the wanted chain length and unsaturation profile.

Fatty acid elongation occurs in cycles of four reactions catalyzed by distinct endoplasmic reticulum-anchored enzymes [8]. In fungi and mammals, after being synthesized in cytosol by a fatty acid synthase (FAS), C16 or C18 fatty acyl-CoA, are transferred to endoplasmic reticulum to be further elongated. First, a condensing enzyme with ketoacyl-CoA synthase activity decarboxylates and condenses a malonyl-CoA to the acyl chain to form a 3-ketoacyl-CoA. Newly formed ketoacyl-CoA is next reduced through the ketoacyl-CoA reductase (KCR) enzyme, then dehydrated by a hydroxyacyl-CoA dehydratase (HCD), before finally being reduced again by an enoyl-CoA reductase (ECR) to obtain an acyl chain with two additional carbons. In eukaryotes, the condensation reaction is carried out by the elongation-defective-like (ELO) enzymes family, except in plants which use the ketoacyl-CoA synthase (KCS) family. These condensing enzymes are generically referred to fatty acid elongases (ELOs) and have been shown to be rate-limiting and responsible for the substrate specificity of the elongation reaction [9, 10]. Organisms typically possess several elongases having distinct specificities for chain lengths or for the number and position of unsaturations. For example, the baker’s yeast *Saccharomyces cerevisiae* has three elongases that are active on saturated fatty acids: ScELO1 elongates from C14 to C18, ScELO2 from C16 to C22 and in a lesser extent C24, while ScELO3 elongates from C18 to C26 [11, 12]. Humans possess seven ELOVL isoforms: HsELOVL1, 3, 6 and 7 act on saturated and monounsaturated fatty acids, with preferences spanning from C16 to C28 [13–16]; HsELOVL2 and HsELOVL5 elongate poly-unsaturated substrates [17]; and HsELOVL4 is active on chains lengths above C26 [18, 19]. Mutations in *ELOVL4* are associated with Stargardt-like macular dystrophy [19], and defects in *ELOVL1* are linked to neurological disorders such as X-linked adrenoleukodystrophy [20]. Accurately identifying substrate specificities of elongases is thus essential for elucidating VLCFA biosynthesis, its roles in human health and to develop efficient chassis for biomanufacturing.

Fatty acid elongases, like many enzymes acting on fatty acids, are membrane-bound, making their in vitro characterization difficult, time-consuming and costly, and potentially prone to artifacts that do not reflect their in vivo activity. One of the most widespread strategies is the deletion of the targeted elongase gene directly in the host genome and the assessment of the deletion effect. This approach can be used to simulate the effect of a deleterious mutation in the context of an elongase-related disease, and to understand the physio-pathological mechanisms involved [21–23]. However, for organisms involving multiple elongases with functional redundancy, the impact of deletion may be partially masked, impeding fine characterization of the enzyme under investigation. Furthermore, native host-based studies are often constrained by the availability of genetic tools and are not applicable to elongases originating from uncultivable organisms or metagenomic sources. To circumvent these limitations and reduce background activity from redundant enzymes, heterologous expression has become a widely adopted approach. *Saccharomyces cerevisiae* is the most commonly used host for such studies due to its genetic tractability and well-characterized endogenous elongation system [24–26]. Other yeasts, including *Pichia pastoris* and *Schizosaccharomyces pombe*, have also been employed for elongase characterization [27, 28]. However, although these yeasts are convenient platforms for functional studies, these validated enzymes need usually to be transferred to another chassis better suited for larger scale fatty acid production.

To develop efficient microbial cell factories for fatty acid production, oleaginous yeasts such as *Rhodotorula toruloides*, *Cutaneotrichosporon oleaginosum*, and *Yarrowia lipolytica* are commonly used due to their natural capacity to accumulate lipids [29, 30]. Characterizing heterologous enzymes directly in these hosts has a dual benefit as it facilitates their subsequent integration into optimized production strains. Among oleaginous yeasts, *Y. lipolytica* stands out as a particularly attractive chassis : its lipid metabolism has been extensively studied, and a wide array of genetic tools is available, two key assets for the functional characterization of heterologous enzymes [31, 32]. However, its native fatty acid profile is relatively narrow, consisting mainly of C16–C18 fatty acids with 0 to 2 unsaturations, and containing only few percent of VLCFAs. *Y. lipolytica* has nonetheless already been successfully engineered to produce a variety of unusual fatty acids, including odd-chain fatty acids, ricinoleic acid, and conjugated linoleic acid [33–35]. Moreover, several VLCFAs, such as erucic acid and nervonic acid, have been produced in engineered strains, and some polyunsaturated like eicosapentaenoic acid (EPA) and docosahexaenoic acid (DHA) have even been produced at commercial scale, highlighting the strong potential of *Y. lipolytica* as both a platform for enzyme characterization and a chassis for tailored fatty acid production [6, 36, 37].

In this study, we developed tools and methods to harness *Yarrowia lipolytica* as a chassis strain for the heterologous characterization of fatty acid-active enzymes, which can then be readily used to develop cell factories. We focused on fatty acid elongases, which are used to produce very long-chain fatty acids. After precise characterization of the two native ELOs from *Yarrowia lipolytica*, we addressed several limitations to increase the characterization throughput. First, we developed a convenient method for strain construction, allowing targeted integration into several genetic backgrounds, where the screening of clones that have integrated the cassette has been facilitated by fluorescence. Next, we miniaturized the strain cultures in 96-well plates, to allow to multiply the culture conditions and evaluate specificity on exogenous substrates added to the medium. To facilitate the quantification and analysis of fatty acids, we then adapted the direct transmethylation protocol in a 96-well format. Finally, we applied this workflow for the simultaneous characterization of the seven human elongases on endogenous fatty acid substrates and on six exogenous PUFAs to demonstrate the efficiency of our improved characterization method from strain construction to fatty acid analysis.

## Materials and methods

### Strains and media

*Escherichia coli* XL1-Blue strain (Agilent, USA) was employed for both cloning and plasmid production. Competent cells were prepared using the Mix & Go Competent Cells kit (Zymo Research, Germany), and transformation was carried out according to the instructions provided by the kit manufacturer.

All *Yarrowia lipolytica* strains used in this study were derived from the wild-type strain W29 (Table 1). *Y. lipolytica* strains were cultured at 28 °C in Yeast extract Peptone Dextrose (YPD) medium for precultures and cell maintenance. A minimum yeast nitrogen base medium (YNB) consisting of 10 g/L glucose, 1.7 g/L YNB without amino acids and ammonium sulfate, 5 g/L NH_4_Cl and 50 mM Na/K phosphate buffer at pH 6.8, was used to select colonies based on prototrophic status. Solid media were prepared by adding 15 g/L agar to the medium. For growth and production, YT_2_D_5_ medium was used. This medium was composed of 10 g/L yeast extract, 20 g/L bacto-tryptone, 50 g/L glucose and 50 mM Na/K phosphate buffer at pH 6.8. Fatty acid stock emulsions at 100 g/L were prepared by sonication of pure fatty acid in water with 0.5% TERGITOL (NP40S, Sigma) as emulsifier.

The yeast cells were made competent and were transformed using the Frozen-EZ Yeast Transformation II Kit (Zymo Research, Germany). Transformants were selected on YPD or YNB agar plates supplemented with 250 µg/mL nourseothricin sulfate (Jena Bioscience, Germany).

**Table 1:** *Y. lipolytica* strains used in this study

Strain name	Parent strain	Genotype	Genetic modification
CyPo6	Po1d	pox1-6∆, *ura3-*,* leu2-*	*Described in Borsenberger et al.*,* 2020* [38]
yPF^−−^	CyPo6	pox1-6∆, fad2∆, *ura3-*,* leu2-*	*YlFAD2 excision using CRISPR*
yPF	yPF^−−^	pox1-6∆, fad2∆, C3::(URA3, LEU2)	*Targeted insertion of auxotrophic markers*
yPF-YlELO1	yPF^−−^	D1::(*URA3*, *pTEF*-*YlELO1*-*tLIP2*, *LEU2*), pox1-6∆, fad2∆	*Targeted insertion by CRISPR*
yPF elo1∆	yPF	elo1∆, pox1-6∆, fad2∆, C3::(URA3, LEU2)	*YlELO1 inactivation (frameshift) using CRISPR*
yPF-YlELO2	yPF^−−^	D1::(*URA3*, *pTEF*-*YlELO2ex*-*tLIP2*, *LEU2*), pox1-6∆, fad2∆	*Targeted insertion by CRISPR*
yPF-HsELOVL7	yPF^−−^	D1::(*URA3*, *pTEF*-*HsELOVL7*-*tLIP2*, *LEU2*), pox1-6∆, fad2∆	*Targeted insertion by CRISPR*
yPF-HsELOVL7 elo2∆	yPF-HsELOVL7	D1::(*URA3*, *pTEF-HsELOVL7-tLIP2*, *LEU2*), *elo2*∆, pox1-6∆, fad2∆	*YlELO2 excision using CRISPR*
yPFK	yPF	pox1-6∆, fad2∆, ku70∆, C3::(URA3, LEU2)	*Ku70 excision using CRISPR*
yPFK-YlELO1	yPFK	D1::(*pTDH-DsRed-tTEF*, *pTEF-YlELO1-tLIP2*), pox1-6∆, fad2∆, ku70∆, C3::(URA3, LEU2)	*Targeted insertion by CRISPR*
yPFK-YlELO2	yPFK	D1::(*pTDH-DsRed-tTEF*, *pTEF-YlELO2-tLIP2*), pox1-6∆, fad2∆, ku70∆, C3::(URA3, LEU2)	*Targeted insertion by CRISPR*
yPFK-HsELOVL7	yPFK	D1::(*pTDH-DsRed-tTEF*, *pTEF-HsELOVL7-tLIP2*), pox1-6∆, fad2∆, ku70∆, C3::(URA3, LEU2)	*Targeted insertion by CRISPR*
yPFK-HsELOVL7 elo2∆	yPFK	ELO2::(*pTDH-DsRed-tTEF*, *pTEF-HsELOVL7-tLIP2*), pox1-6∆, fad2∆, ku70∆, C3::(URA3, LEU2)	*Simultaneous YlELO2 deletion and targeted cassette insertion using CRISPR*
yPFK-HsELOVL1	yPFK	D1::(*pTDH-DsRed-tTEF*, *pTEF-HsELOVL1-tLIP2*), pox1-6∆, fad2∆, ku70∆, C3::(URA3, LEU2)	*Targeted insertion by CRISPR*
yPFK-HsELOVL1 elo2∆	yPFK	ELO2::(*pTDH-DsRed-tTEF*, *pTEF-HsELOVL1-tLIP2*), pox1-6∆, fad2∆, ku70∆, C3::(URA3, LEU2)	*Simultaneous YlELO2 deletion and targeted cassette insertion using CRISPR*
yPFK-HsELOVL2	yPFK	D1::(*pTDH-DsRed-tTEF*, *pTEF-HsELOVL2-tLIP2*), pox1-6∆, fad2∆, ku70∆, C3::(URA3, LEU2)	*Targeted insertion by CRISPR*
yPFK-HsELOVL3	yPFK	D1::(*pTDH-DsRed-tTEF*, *pTEF-HsELOVL3-tLIP2*), pox1-6∆, fad2∆, ku70∆, C3::(URA3, LEU2)	*Targeted insertion by CRISPR*
yPFK-HsELOVL3 elo2∆	yPFK	ELO2::(*pTDH-DsRed-tTEF*, *pTEF-HsELOVL3-tLIP2*), pox1-6∆, fad2∆, ku70∆, C3::(URA3, LEU2)	*Simultaneous YlELO2 deletion and targeted cassette insertion using CRISPR*
yPFK-HsELOVL4	yPFK	D1::(*pTDH-DsRed-tTEF*, *pTEF-HsELOVL4-tLIP2*), pox1-6∆, fad2∆, ku70∆, C3::(URA3, LEU2)	*Targeted insertion by CRISPR*
yPFK-HsELOVL5	yPFK	D1::(*pTDH-DsRed-tTEF*, *pTEF-HsELOVL5-tLIP2*), pox1-6∆, fad2∆, ku70∆, C3::(URA3, LEU2)	*Targeted insertion by CRISPR*
yPFK-HsELOVL6	yPFK	D1::(*pTDH-DsRed-tTEF*, *pTEF-HsELOVL6-tLIP2*), pox1-6∆, fad2∆, ku70∆, C3::(URA3, LEU2)	*Targeted insertion by CRISPR*

### General molecular biology techniques

PCRs were performed on a T100 thermal cycler (BioRad). The “CloneAmp HiFi PCR Premix” (Takara Bio) kit was used according to the supplier’s recommendations. The template DNA could be purified DNA, or it could be obtained directly from colonies. For PCR on *Yarrowia lipolytica* colonies, a small amount of biomass was resuspended in 25 µL of 20 mM NaOH and incubated at 95 °C for 10 min. The supernatant was recovered after centrifugation and contained the template DNA for PCR. Typically, 1 µL was used for 25 µL PCR.

Plasmids for CRISPR-Cas9 components expression (pCg series, Figures S1 and S2) were constructed as described previously [38, 39]. Plasmids carrying recombination templates for targeted gene integration by CRISPR-Cas9-induced homologous recombination (pHR series, Figure S3) were constructed by InFusion cloning (Takara Bio, Japan). The building blocks for plasmid assembly were generated by PCR amplification on either previously constructed plasmids or genomic DNA from the Po1d strain. Heterologous genes coding for fatty acid elongases were codon-optimized for *Y. lipolytica* and synthesized by Genecust (France). Endogenous genes YlELO1 (YALI1_F09932g) and YlELO2 (YALI1_B26350g) were amplified from genomic DNA. All gene sequences and accession numbers are provided in Table S1.

### Targeted gene insertion using CRISPR-Cas9

Targeted gene insertion was performed using a replicative pCg71 plasmid expressing all CRISPR components and a sgRNA targeting the integration site such as C3 and D1 [40], co-transformed with a PCR-amplified linear recombination template containing the ELO expression cassette flanked by two 1 kb sequences homologous to each side of the targeted cut site. Linear templates were amplified from pHR plasmids by PCR in 100 µL of final reaction using CloneAmp HiFi PCR Premix (Takara Bio, Japan). PCR products were purified on a single column using the Macherey-Nagel NucleoSpin Gel and PCR Clean-Up kit and eluted in 20 µL elution buffer. This led to a final DNA concentration of the linear recombination template ranging from 300 to 500 ng/µL. A transformation of 25 µL competent cells was performed by adding 2 µL of purified linear template (600–1000 ng total) and 0.5 µL of pCg71 plasmid (50 ng). 250 µL of EZ-3 solution was added before a 50 min incubation at 28°C. After incubation, cells were plated on agar plates supplemented with nourseothricin to select clones that have integrated the pCg71 plasmid. After the first selection, a second selection was performed to select clones having integrated the cassette into the genome. To facilitate this secondary screening, we developed two types of recombination cassettes depending on the secondary screening.

**Fig. 1:**
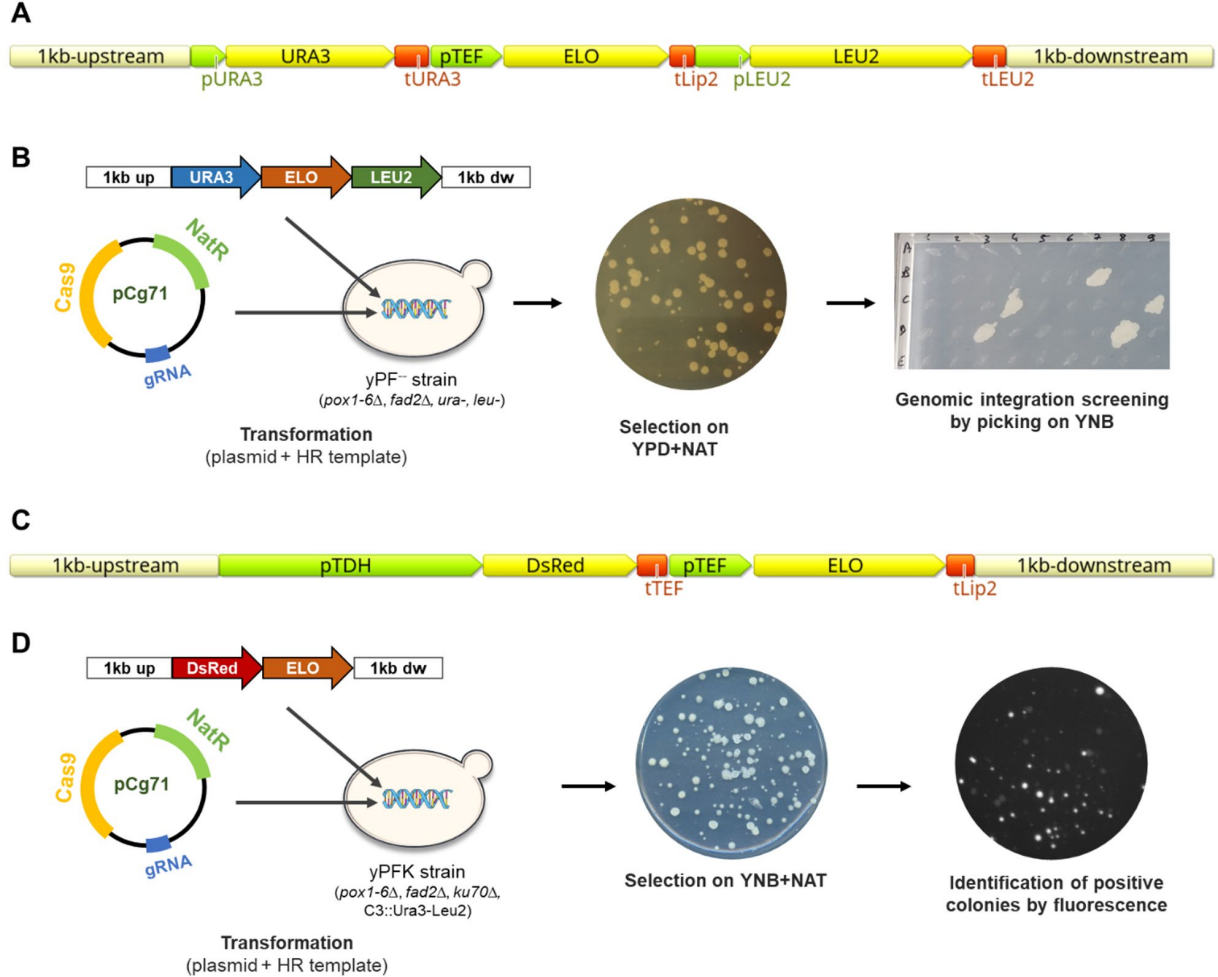
Targeted gene insertion strategies using CRISPR-mediated homologous recombination. **A** Generic recombination template for integration of ELO sequence using auxotrophic markers for secondary selection screening of integration into genome. **B** Main steps for the generation of yPF ELO-expressing strains using auxotrophic screening. **C** Generic recombination template for integration of ELO cassette using fluorescence screening for integration into genome. **D** Main steps for the generation of yPFK ELO-expressing strains using fluorescence screening

In the first strategy, URA3 and LEU2 auxotrophic markers were flanked around the ELO expression cassette (pTEF-ELO-tLip2) to allow a second round of selection by auxotrophy (Fig. 1A–B). Transformation is performed in yPF^−−^ strain (Ura-, Leu-) as described above. The resulting nourseothricin-resistant clones were then transferred on YNB medium for secondary screening on genome integration to select prototrophic strains that have integrated the cassette. After plasmid curation with a 24 h cultivation in non-selective YPD medium genomic DNA from prototrophic clones was extracted using the YeaStar Genomic DNA Kit (Zymo Research, Germany). Correct insertion at locus was finally validated by PCR on gDNA.

In our second improved strategy, the ELO expression cassette was integrated together with a pTDH-DsRed-tTEF cassette that allows the expression of the fluorescence protein DsRed (Fig. 1C–D). Transformation were performed in yPFK strain (Ura+, Leu+) as described above, but as YPD medium is autofluorescent, the cells were plated on a YNB + NAT medium for the first selection. Fluorescent colonies were identified by visualizing the transformation plates on a ChemiDoc imager (BioRad, France) using « Cy3 Blot (602/50, Green Epi) » preset. Fluorescent colonies were then re-isolated by streaking on new fresh YNB + NAT plates and re-visualized by fluorescence to ensure the absence of mosaicism. Correct insertion at locus was finally verified by colony PCR.

Strain yPF was constructed as a control to compare fatty acid profiles of the different prototrophic strains constructed. URA3 and LEU2 markers were integrated in yPF^−−^ strain at neutral C3 locus following the same procedure as described above.

Guide sequences for integration in locus C3 (gIntC3), D1 (gIntD1) or ELO2 (gIntELO2), as well as primers for template amplification and for the verification of cassette integration at locus are given in Table S2 and Table S3.

### Gene deletion by CRISPR-Cas9

All gene inactivation relied on CRISPR-Cas9 plasmids and methods developed and described by Borsenberger et al. [38, 39]. Briefly, approx. 100 ng of pCg71 (one guide) or pCg72 plasmid (two guides) were mixed with 25 µL competent cells. Transformation mixture was spread on YPD+NAT for transformants selection. Individual colonies were then spread on a new YPD+NAT plate.

Due to its essential role, deletions of YlELO2 gene (YALI1_B26350g) were performed on strains in which another elongase had previously been integrated. The gene was inactivated using two guides targeting the intron and the terminator, spaced 1602 bp apart. Large deletion or inversion of the sequence between the two cut sites were screened by colony PCR and sequencing. Ku70 gene (YALI1_C11925g) was inactivated similarly in yPF strain using two guides spaced 899 bp apart on the coding sequence. YlELO1 gene (YALI1_F09932g) was inactivated in yPF using one guide targeting 84 bp downstream the start codon. Frameshift mutation were screened by colony PCR and sequencing.

Guide sequences, as well as primers for verification of deletions are given in Table S2 and Table S3.

### Standard protocol for cultures and fatty acid analysis

Isolated clones freshly grown on plates were inoculated for preculture in 3 mL YPD for 24 to 48 h at 28 °C with 140 rpm agitation. Cultures were then inoculated at OD 0.5 into 100 mL baffled flasks filled with 15 mL YT_2_D_5_. Cultures were incubated for 48 h at 28 °C with 140 rpm shaking. At the end of the cultivation, the final OD at 600 nm was measured, then 1 mL of the culture broth was transferred to 7 mL glass vial and was freeze-dried for fatty acid extraction.

To be quantified by gas chromatography (GC), total fatty acids must be extracted from the various lipid pools and derivatized to form fatty acid methyl esters (FAME). Transmethylation protocol was adapted from Robin et al. [41]. About 2 mL of methanol solution containing 2.5% sulfuric acid (V/V) and 0.5 g/L heptadecanoic acid (C17:0) as an extraction standard was added to 2 mL freeze-dried cell culture. After incubating for 4 h at 80 °C with periodic mixing, the samples were cooled to room temperature. Subsequently, 1.5 mL of 50 g/L NaCl solution and 2 mL of decane containing 0.2 g/L mC12:0 as an internal standard were added to each sample. Following thorough mixing, the samples were centrifuged at 2,000 g for 2 min. The organic upper phase obtained was then collected for GC-FID/MS analysis.

### Miniaturized protocol for cultures and fatty acid analysis

Isolated clones freshly grown on plates were inoculated for preculture in 3 mL YPD for 24 to 48 h at 28 °C with 140 rpm agitation. Cultures were then inoculated at OD 0.5 into 96-well deepwell plates (AB-0661, ABgene, Thermo Scientific) filled with 500 µL of YT_2_D_5_. Exogenous fatty acids could be added to medium at a final concentration of 1 g/L from 100 g/L stock emulsion. Plates were covered with breathable sealing tape (Corning, Netherlands), and incubated for 48 h at 28 °C with 800 rpm shaking in Microtron incubator with 3 mm throw (Infors HT, France). At the end of the cultivation, plates were centrifuged 5 min at 3700 rpm to allow supernatant to be removed by flicking the plates. Plates with cell pellets were then freeze-dried for 24 h.

Fatty acid extraction and transmethylation from freeze-dried cells were adapted from the standard protocol, with some modifications to fit the 96-well plate format. About 500 µL of methanol solution containing 2.5% sulfuric acid (V/V) and 0.5 g/L heptadecanoic acid (C17:0) as an extraction standard was added in each well. Plates were tightly closed by thermal sealing with ALPS 50 V manual thermocellor (Thermo Scientific) at 200 °C for 6 s using Easy Pierce Foil (AB-0757, ABgene, Thermo Scientific). After incubating for 24 h at 60 °C, the plates were cooled to room temperature. Subsequently, 350 µL of 50 g/L NaCl solution and 500 µL of decane containing 0.2 g/L mC12:0 as an internal standard were added to each well. Following cautious but vigorous mixing by up and down pipetting, the plates were centrifuged at 2000 g for 2 min. The organic upper phase obtained was then collected for analysis by GC-FID/MS.

### Gaz chromatography for FAME quantification

Most of the FAME quantifications were performed using a GC-FID/MS (Thermo Fisher Scientific, Trace 1310-ISQ LT) with DB-5MS column (Thermo Fisher Scientific, 30 m x 0.32 mm x 0.25 μm) using hydrogen as carrier gas with constant flow set at 5 mL/min. 1 µL of sample was injected at 250 °C with a split ratio 1:5. FID detector was set at 330 °C. Quantification was performed using FID signal using various FAME calibration solutions in decane. FAME used for calibration were purchased from NuChek Inc, USA. Two temperature programs were used for FAME separation. Program 1 (Table S4A), used for samples without PUFA addition, was the fastest. Program 2 (Table S4B) was used for samples containing exogenous PUFA addition, except for C18:3ω3 which co-elutes with C18:1 on this column regardless of program used.

For samples with C18:3ω3 addition, FAME quantification was performed using a GC-FID (HP 6890) with Select-FAME column (Agilent 50 m x 0.25 mm) using nitrogen as carrier gas with constant pressure set at 3.15 bar. 1 µL of sample was injected at 250 °C with a split ratio 1:5. FID detector was set at 300 °C. Oven temperature program is detailed in Table S4C.

### Data analysis

Plasmid design and sequence analysis were carried out using Geneious Prime 2025 (Dotmatics). Peak areas on chromatograms were integrated using Chromeleon software (Thermo Scientific) version 7.2.9 for TRACE1310 GC-FID/MS, version 6.80 for HP-6890 GC-FID. Peak areas in samples were normalized using mC12:0 internal standard, then corrected using C17:0 for incomplete liquid-liquid extraction. Concentration of each fatty acid in samples was calculated from peak area and standard curves.

Differences in fatty acid composition between strains were assessed using PERMANOVA (vegan, adonis2) on Bray–Curtis dissimilarities calculated from relative abundances. The Hellinger transformation was applied to reduce the influence of dominant fatty acids and stabilize variance across samples. The effect of strain on multivariate profiles was tested with 999 unrestricted permutations, and significance was determined from R², pseudo-F, and permutation-based P values. Homogeneity of dispersions was verified using PERMDISP, and pairwise PERMANOVA comparisons were adjusted for multiple testing (Benjamini–Hochberg). Multivariate patterns were visualized via PCoA with 95% confidence ellipses, and the contribution of individual fatty acids to ordination patterns was evaluated using vector fitting (envfit).

## Results

### Characterization of *Yarrowia lipolytica*’s native ELOs

**Fig. 2:**
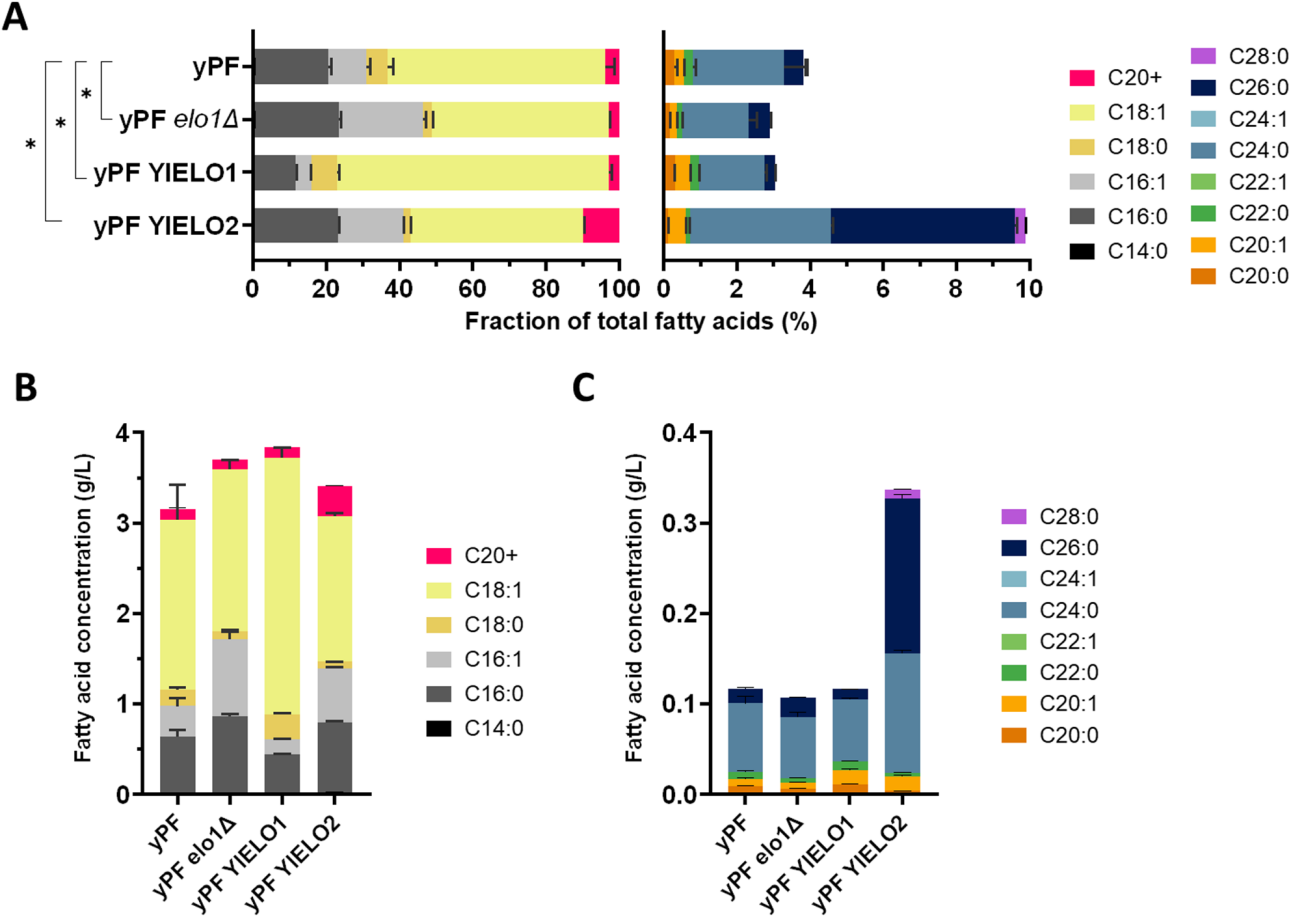
Impact of overexpression of the two *Y. lipolytica *natives elongases on the fatty acid profile of the yPFK strain. The fatty acid content of strains whose elongases have been inactivated or overexpressed was analyzed by GC-FID/MS. The data represent the mean ± standard deviation (*n* = 4). **A** Fatty acid profiles represented as fraction of total fatty acids (TFA). Left panel shows the main fatty acids (C14-C18), while the right panel shows an enlargement of the VLCFA (C20+) fraction. Statistically significant difference of fatty acid profile with the baseline strain yPF is denoted (*) p-value < 0.05 (pairwise PERMANOVA test). **B–C** Fatty acid content expressed as concentration in culture medium

Before characterizing heterologous ELOs, it is necessary to characterize first the specificity of endogenous elongation enzymes. *Yarrowia lipolytica* has been described as possessing two ELOs (YlELO1 and YlELO2), which have previously been characterized in our lab using knock-out mutants as being active on saturated fatty acids. YlELO1 (YALI1_F09932g) is responsible for the elongation of C16 to C18, while YlELO2 (YALI1_B26350g) is involved in the synthesis of VLCFAs [42]. To complete this preliminary work, we overexpressed these two endogenous enzymes in *Y. lipolytica*. The yPF strain, inactivated for beta-oxidation (POX1-6 genes) and for the Fad2 desaturase, was selected as a chassis to prevent degradation of elongated products and to maximize elongation of saturated and monounsaturated fatty acids (SFA and MUFA). The encoding sequences for the two elongases under the control of the strong constitutive pTEF promoter were integrated by CRISPR-Cas9-mediated homologous recombination into the neutral D1 locus of the yPF strain. URA3 and LEU2 selection markers flanking the expression cassette allow a secondary selection of clones having integrated the cassette by homologous recombination rather than having undergone non-homologous end-joining repair. We also inactivated YlELO1 by introducing a frameshift mutation by CRISPR-Cas9, but despite numerous trials, we were not able to inactivate YlELO2 confirming that it is an essential gene. The fatty acid profiles of the strains were analyzed after 48 h of culture in YT_2_D_5_-rich medium (Fig. 2, Table S5).

The fatty acid profile of the yPF strain without overexpression is characteristic of *Yarrowia lipolytica* inactivated for ∆12-desaturation. The main fatty acids produced are as follows: C18:1 (59.4% of total fatty acids, TFA), C16:0 (20.1%), C16:1 (10.5%) and C18:0 (5.9%). Fatty acids with chain lengths of C20 and longer represent only 3.8% of the total and 0.12 g/L, with C24:0 accounting for the majority of these (65.3% of total VLCFA). The longest chains produced are C26:0 for saturated fatty acids and C20:1 (elongation of C18:1) for monounsaturated fatty acids. Overexpression of YlELO1 resulted in an increase in the proportion of C18s and a decrease in C16s. The fraction of VLCFAs is not altered. On the contrary, YlELO1 deletion resulted in a decrease in C18s while C16s increased. When YlELO2 is overexpressed, VLCFA fraction increased to 9.9% of TFA and 0.34 g/L, driven by an increase in C24:0 (3.9%), C26:0 (5.0%) and a new fraction of C28:0 (0.3%). The main fatty acid produced is the C26:0 which represents 50.8% of the total VLCFA. Conversely, the proportion and quantity of C16s increased, while C18s decreased. The results obtained confirm and refine the previous characterization [42], showing that YlELO1 is indeed responsible for the elongation of C16:0 to C18:0, while YlELO2 is involved in the elongation of saturated fatty acids up to C24:0 and C26:0. Both enzymes also appear to be involved in the elongation of oleic acid in C20:1 as a small increase is observed for each strain. To further examine relationships between strains, a PCoA based on Bray–Curtis distances was performed (Figure S4). The four strains formed well separated clusters, consistent with their distinct fatty acid profiles. The envfit projection of individual fatty acids indicated that variation along the primary axis was largely driven by very long chain saturated fatty acids (C24:0, C26:0 and C28:0), which strongly oriented in the direction of the YlELO2 overexpression strain. In contrast, the opposite direction of this axis was associated with higher proportions of C16:0 and C16:1, while separation along the secondary axis was mainly influenced by C18:0 and C18:1. These patterns support the biochemical interpretation that YlELO1 primarily modulates the balance between C16 and C18 species, whereas YlELO2 drives the accumulation of VLCFAs.

### Characterization of human ELOVL7 in *Yarrowia lipolytica*

**Fig. 3:**
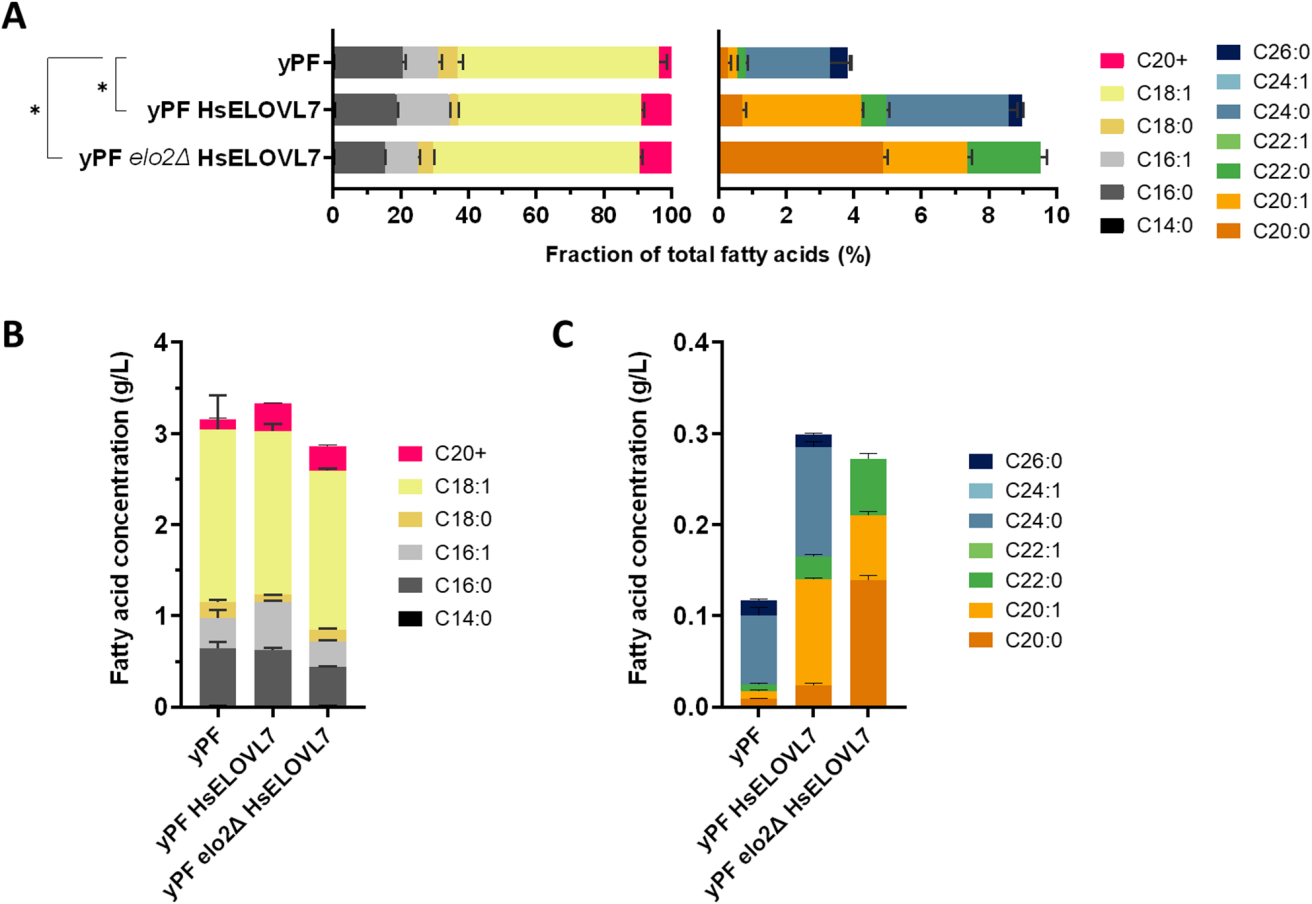
Impact of HsELOVL7 expression on the fatty acid profile in a YlELO2-deleted background. Fatty acid content of strain expressing HsELOVL7, inactivated (*elo2∆*) or not for the YlELO2 was analyzed by GC-FID/MS. The data represent the mean ± standard deviation (*n* = 4). **A** Fatty acid profiles represented as fractions of total fatty acids (TFA). Left panel shows the long chain fatty acids (C14-C18), while the right panel shows an enlargement of the VLCFA (C20+) fraction. Statistically significant difference of fatty acid profile with the baseline strain yPF is denoted (*) p-value < 0.05 (pairwise PERMANOVA test). **B–C** Fatty acid content expressed as concentration in culture medium

Given that the strategy of overexpressing endogenous ELOs in *Yarrowia lipolytica* enabled to successfully characterize their substrate specificity, we decided to apply a similar strategy to the human fatty acid elongase HsELOVL7. This elongase was described as being able to elongate saturated fatty acids up to C22 [43]. The *Yarrowia lipolytica* codon-optimized coding sequence was integrated into the yPF^−−^ strain at D1 locus to construct the overexpression strain, whose fatty acid profile was analyzed **(**Fig. 3 and Table S6).

Expression of HsELOVL7 induced an increase in the total % of VLCFA going from 3.8% in the YPF strain to 9.0% in the strain expressing the human elongase. We also observed a marked increase in C20:1 (from 0.3 to 3.5%) and a moderate increase in C20:0 (from 0.3 to 0.7%), C22:0 (from 0.2 to 0.8%) and C24:0 (from 2.5 to 3.6%). The proportion of C26:0 remains similar. Despite these findings, the specificity of HsELOVL7 for saturated fatty acids is not completely clear. Since the native YlELO2 elongase responsible for elongation up to C26:0 is still present in the chassis, the substrate specificity of the heterologous ELO is difficult to determine without any ambiguity. As YlELO2 is an essential gene, it is therefore impossible to integrate the expression cassette into a yPF *elo2∆* strain, so it is necessary to proceed sequentially with the deletion of native ELO2 after integration of another elongase that can complement.

The native YlELO2 gene was thus deleted in the yPF-HsELOVL7 strain by CRISPR-Cas9. Two guides spaced 1602 bp apart were used to drive a large deletion of the fragment between the two cut sites. The *elo2∆* strain was obtained and validated by sequencing, confirming that HsELOVL7 is capable of complementing VLCFA synthesis. In total, VLCFAs represent 9.5% of TFAs. The fatty acid profile of the strain was modified accordingly: saturated fatty acid elongation now stops at C22:0 (2.2% of TFA), with a large proportion of C20:0 (4.9%) representing 51.0% of VLCFAs **(**Fig. 3, Table S6). The elongation of MUFAs does not seem to be affected compared with the non-deleted strain, with C20:1 being the longest monounsaturated accounting for 2.5% of TFA. The PCoA also showed clear separation between yPF, yPF HsELOVL7 and yPF *elo2Δ* HsELOVL7, consistent with their altered VLCFA profiles (Figure S5). Envfit vectors indicated that C20:0, C22:0 and C20:1 were the main contributors to strain separation. These results highlight the necessity of deleting the native YlELO2 to avoid functional redundancy and thus to characterize accurately the activity of heterologous ELOs on SFAs and MUFAs.

### Accelerate the strain construction: simultaneous knock-out of the endogenous YlELO2 and knock-in of the heterologous ELO genes

**Fig. 4:**
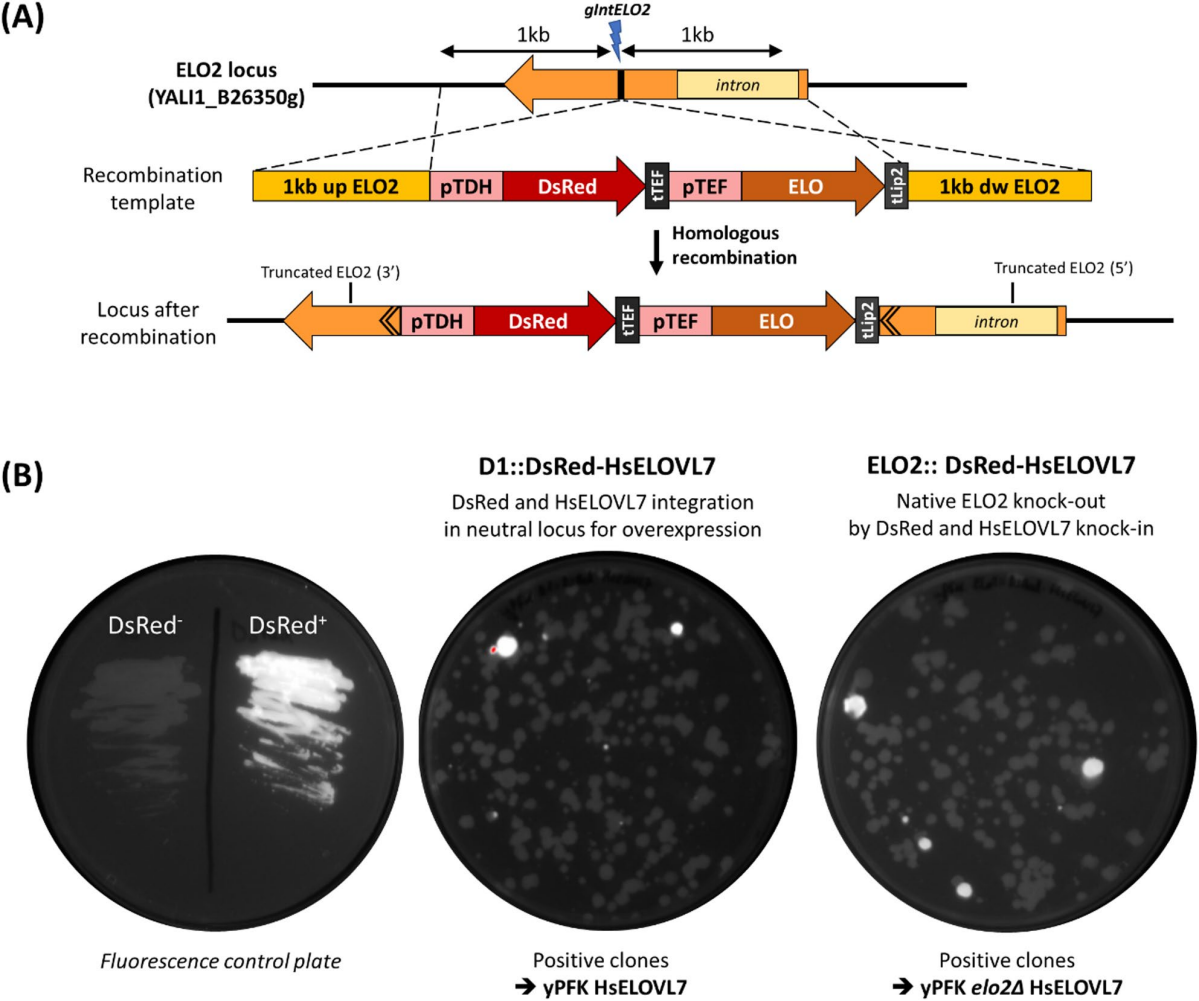
Strain engineering strategy developed to characterize heterologous elongases. **A** Generation of the *elo2∆* strain using a YlELO2 Knock-Out via heterologous ELO Knock-In approach. Native gene is targeted by CRISPR-Cas9, and recombination template is used to integrate by homologous recombination a tandem cassette DsRed-ELO, leading to disrupted YlELO2. **B** Transformation plates resulting from the integration of tandem DsRed-HsELOVL7 cassette into D1 or ELO2 locus. Left plate was inoculated with DsRed^-^ or DsRed^+^ strain as fluorescence standard. As the transformation plates contain small fluorescent colonies, enlarged pictures are shown in Figure S7 for better visibility

With the experiments described above, we proved that it was necessary to delete the native elongase YlELO2 to evaluate the specificity of heterologous ELOs in *Y. lipolytica*. But as this gene is essential, we had to construct the strain in two steps by (1) first integrating the heterologous elongase, (2) then deleting the YlELO2 gene. This two-step method is time-consuming and requires extensive screening to check both integration and deletion events and would be suitable to evaluate only a reduced number of elongases. To increase screening throughput of the method, we developed a new one-step construction system based on YlELO2 knock-out by heterologous ELO knock-in (Fig. 4A**)**.

Moreover, to further increase the throughput and decrease screening efforts, the Ku70 gene was deleted in the yPF strain, leading to the yPFK strain. This gene is involved in NHEJ repair and its deletion has been shown to promote homologous recombination. Then, an RNA guide designed to target YlELO2 coding sequence (intELO2) was evaluated by expressing it (without homologous recombination template) in yPF-HsELOVL7 strain, and it showed excellent efficiency (Figure S6).

Because this guide targets an essential gene, we expected that transformation of the CRISPR plasmid into an uncomplemented strain would not allow to obtain transformants due to a lethal effect. Surprisingly, the transformation led to the growth of colonies in a similar number to those obtained with pCg71-intD1 (~ 200 colonies per 25 µL of cells). Sequencing revealed that all clones out of the 11 tested are mutation-free in the YlELO2 gene. This suggests that by an unknown mechanism, the strain can escape CRISPR-Cas9 double strand break to counteract the lethality caused by the deletion. This background noise of non-edited clones could make the HR integration screening work still labor-intensive.

To overcome this limitation, we decided to co-integrate the ELO expression cassette together with that of the fluorescent protein DsRed substituting the auxotrophic selection markers. This will avoid the replicating step on YNB medium and allow to select fluorescent clones that have inserted the recombination cassette just by exposing transformation plates on a fluorescence visualizer. The DsRed-ELO cassette was integrated in the reverse direction of the YlELO2 gene to circumvent any possible regulation effects by the native promoter.

Tandem constructs for DsRed-HsELOVL7 integration into D1 and ELO2 loci were then transformed into yPFK strain (Fig. 4B and Figure S7). For each construct, five fluorescent clones and three negative ones were transferred to a new plate. After 24 h of growth, fluorescence is still clearly visible on positive clones and can be distinguished from non-fluorescents ones (Figure S8). Colony PCR were performed to check the correct integration in each locus. In all fluorescent clones tested, PCR and sequencing showed that cassette integration by HR in targeted locus successfully occurred. Non-fluorescent clones did not integrate at targeted locus, as expected. Sequencing of targeted locus revealed that non-fluorescent colonies are mutation-free (Figure S9 and Figure S10). Taken together, those results confirm that both the fluorescence screening method and the Knock-Out of ELO2 by Knock-In of heterologous ELO are functional.

We applied the same procedure using YlELO1 in the recombination cassette, that is only able to elongate up to C18. Integration in D1 locus was successfully obtained from the first transformation experiment. However, despite three independent transformation experiments, no fluorescent clone was obtained for integration at YlELO2 locus. As expected, knock-out by knock-in appears only possible when the integrated ELO is able to elongate to form VLCFAs, and thus complement the functionality loss of the essential YlELO2 gene.

Fatty acid profiles of the strains constructed using the new procedure (yPFK strain and screening by fluorescence) were analyzed and showed to be identical to those constructed using the previous method (yPF strain and screening using auxotrophy) (Figure S11).

### Miniaturization of cultures and fatty acid analysis

The described characterization method is limited to endogenous saturated and mono-unsaturated fatty acids present in the chassis strain. To access other fatty acid structures and broaden their characterization, we decided to add exogenous fatty acids to the medium. As it also increases the number of culture conditions, we have developed a miniaturized method for the culture and analysis of fatty acids both to maximize the screening throughput and to minimize the cost of such experiments. The cultures were first miniaturized in 2 mL deepwell plates filled with 500 µL of rich YT_2_D_5_ rich medium, for 48 h at 800 rpm agitation. These parameters enabled similar final OD to those obtained in flasks (between 80 and 140 depending on the strain) to be achieved.

**Fig. 5:**
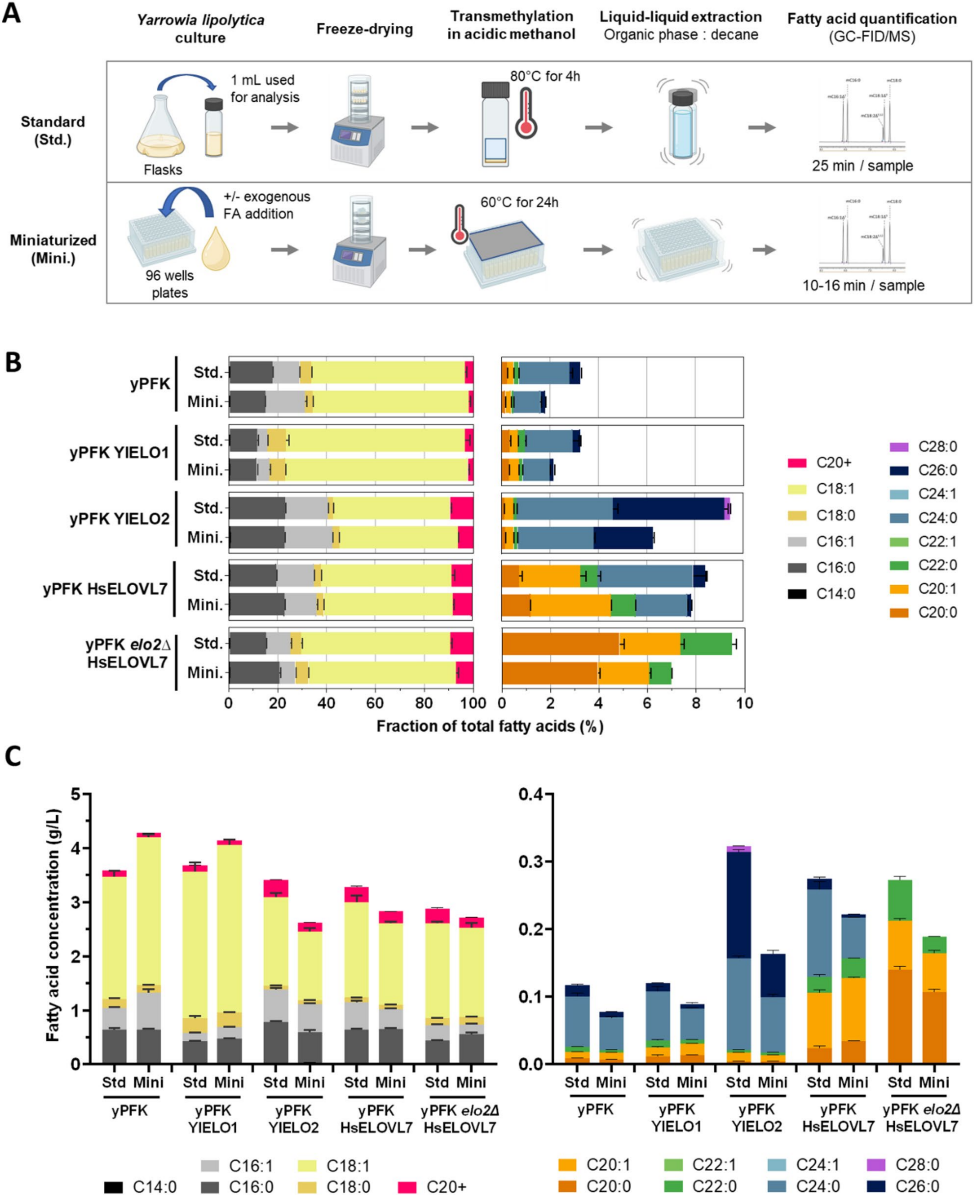
Miniaturization of cultures and fatty acid analysis for the characterization of elongases. **A** Experimental steps for entire workflow were adapted to be performed entirely in 96-well format. **B** Comparison of fatty acid profiles obtained from strains expressing elongases between the standard (Std.) and the miniaturized (Mini.) protocols. Fatty acid content of strains was analyzed by GC-FID/MS. The concentration of each fatty acid was reported relative to the total fatty acid concentration. **C** Fatty acid concentrations expressed in g/L. The data represent the mean ± standard deviation (*n* ≥ 3)

We then transposed the fatty acid extraction and transmethylation method so that it could be carried out in the same deepwell plate used for the cultures (Fig. 5). To prevent evaporation of the methanol reaction solution, the plate was thermally sealed using an aluminum cover and the transmethylation temperature was reduced from 80 to 60 °C, while the reaction time was extended from 4 to 24 h. Liquid-liquid extraction using decane was carried out by mixing by pipetting to avoid contamination between wells. As the extraction yield of fatty acids in the organic phase was lower and variable, C17:0 was added to the acidic methanol as an internal extraction standard to normalize concentrations.

The fatty acid profiles obtained with the new method for cultures and fatty acid analysis were compared to those obtained previously using strains overexpressing native ELOs and those expressing HsELOVL7 (Fig. 5, Table S7). Significant differences (*p* < 0.05) were detected between groups corresponding to the same strain when analyzed using either the standard or the miniaturized protocol. However, the PCoA visualization shows that samples from a given strain cluster closely regardless of the analytical method, while remaining well separated from other strains (Figure S12). This indicates that, despite these statistical differences, strain-specific fatty-acid profiles remain clearly distinguishable with the miniaturized procedure. It should be noted that the concentrations of very long-chain saturated fatty acids with more than 24 carbons are underestimated compared with transmethylation in vials. Indeed, the C24:0 and C26:0 fractions are reduced, and the C28:0 of YlELO2 is no longer even detected, while for all other fatty acids, the proportions found are similar. To determine whether this difference in profile was related to the downscaling of cultures or resulted from the new transmethylation method, the strains were grown separately in flasks and deepwell plates. After cultivation, 500 µL of the flask cultures were transferred to the deepwell, and the samples were then processed and transmethylated together in the deepwell (Figure S13). The profiles obtained are visually very similar for the same strain regardless of the culture method. Although pairwise PERMANOVA reveals significant differences, PCoA representation shows that the groups for the same strain cluster together closely. These results suggest that the difference in profile observed between the two protocols is due to the transmethylation method. We hypothesize that at a lower temperature, such long saturated FAMEs are not fully soluble in the methanol solution, thus limiting the reaction and their subsequent extraction. Nevertheless, the differences in profiles between the strains expressing the different ELOs are clearly visible and representative of the expected specificities. We ensured that the fatty acid profiles obtained were reproducible between several series of cultures and analyses carried out at different times (Figure S14). The new miniaturized method for cultures and fatty acid analysis is effective and robust to characterize the specificity of heterologous ELOs.

### Simultaneous characterization of the seven human ELOVLs using improved workflow from strain engineering to analysis

**Fig. 6:**
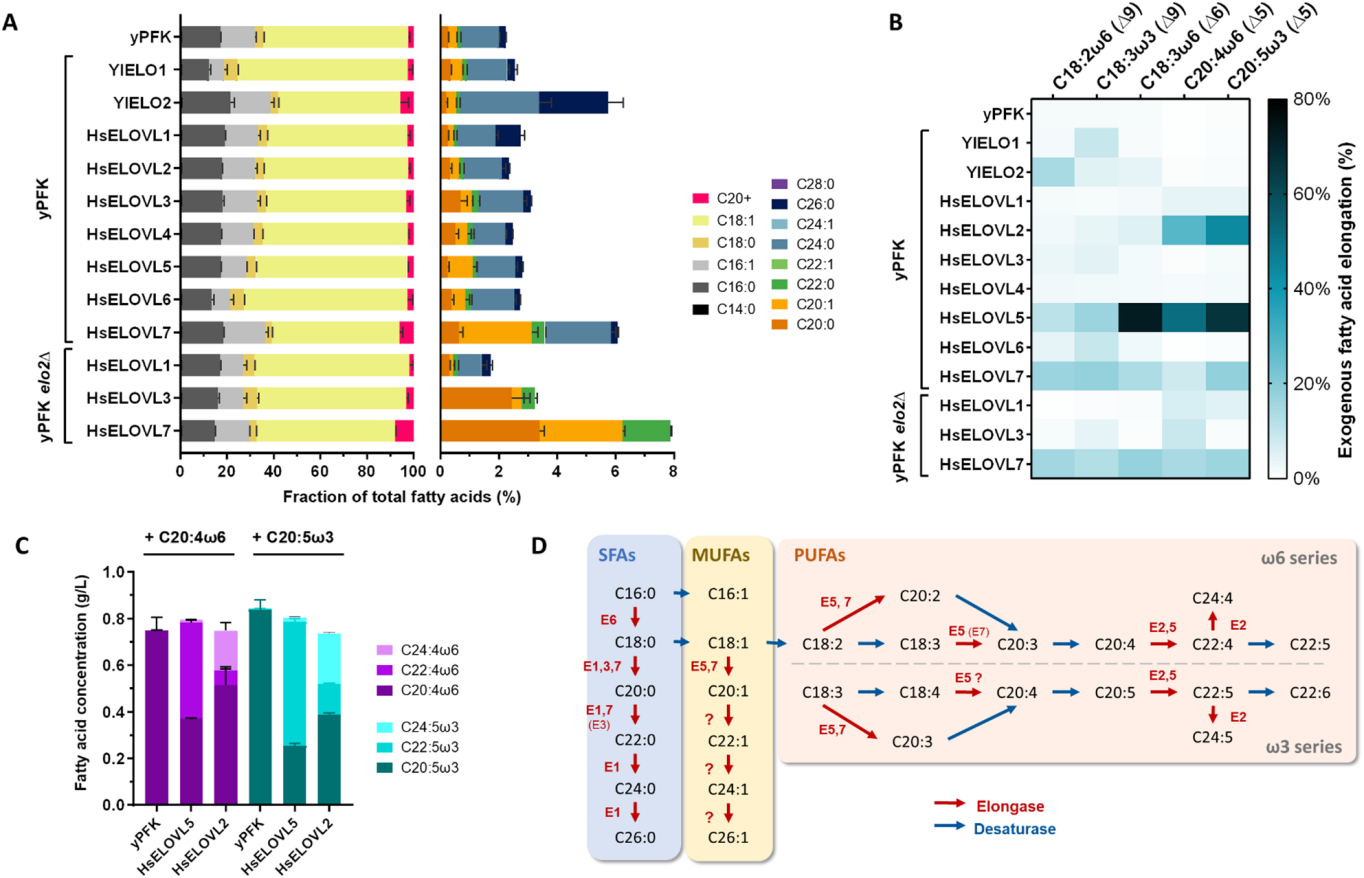
Simultaneous characterization of the seven human ELOVLs and the two native ELOs from *Yarrowia lipolytica*. Fatty acid content of strains was analyzed by GC-FID/MS, using the miniaturized workflow. The data represent the mean ± standard deviation (*n* ≥ 4). **A** Specificity for endogenous saturated and monounsaturated fatty acids. The concentration of each fatty acid was reported relative to the total fatty acid concentration. Rigth panel is an enlargement of C20 + fraction from left panel, representing fractions for each very-long chain fatty acid. **B** Specificity for exogenous polyunsaturated fatty acids. Exogenous fatty acid elongation rate represents the measured molar ratio of elongation product over initial substrate (product + remaining substrate). **C** Human ELOVL2 and ELOVL5 can elongate C20 PUFAs up to C24. **D** Reconstruction of the human VLCFA metabolic pathway involving human ELOVLs based on their specificity characterized in *Yarrowia lipolytica.* Dashed arrow between C18:2 and C18:3ω3 means the desaturase involved is not present in mammals

To demonstrate the strength of the developed method for characterizing ELOs in *Y. lipolytica*, we characterized all seven human ELOVLs in a single series of experiments. The six expression cassettes for HsELOVL1 to HsELOVL6, were transformed into yPFK for integration at either D1 or ELO2 locus. Transformation plates were visualized for fluorescence to discriminate colonies which have integrated the cassette. All human elongase genes could be integrated in D1 locus. However, only HsELOVL1 and HsELOVL3 could be integrated in ELO2 locus. Despite several attempts, we were unable to obtain fluorescent clones for the integration of HsELOVL2, 4, 5 and 6 in ELO2 locus, suggesting these enzymes are not active or not able to complement YlELO2 deletion. The strains thus obtained, as well as those overexpressing the native elongases of *Yarrowia lipolytica*, were cultivated in deepwell in the same batch with or without addition of five exogenous polyunsaturated fatty acids individually. The fatty acids were extracted and transmethylated in a single step using the miniaturized protocol (Fig. 6A–B, Tables S8 and S9). A total of 4 deepwell plates were cultured with each strain in at least 4 replicates. Strains yPFK, yPFK YlELO1, yPFK YlELO2 were added to each deepwell plate as controls to monitor variability between plates.

Concerning endogenous fatty acid elongation, the fatty acid profiles of the control yPFK, YlELO1, and YlELO2 strains are like those obtained previously (Fig. 6A**)**. As expected, the VLCFA profiles of the strains overexpressing human ELOVLs that do not complement YlELO2 (HsELOVL2, HsELOVL4, HsELOVL5 and HsELOVL6) are similar to the control strain except for HsELOVL5 that seems able to elongate C18:1 in C20:1 with a moderate increase in C20:1 (from 0.3 to 0.84% of TFAs). If HsELOVL6 does not cause any change in the VLCFA fraction, the proportion of C18 increases while the proportion of C16 decreases compared to control strain, from 65.76% to 75.88% and from 31.57% to 21.0%, respectively (Table S10). HsELOVL1 mainly produces C24:0 and C26:0, whereas main VLCFAs produced by HsELOVL3 are C20:0 (2.4% of TFAs), C22:0 (0.5%) and C20:1 (0.34%). The profiles obtained for the two HsELOVL7 strains are like those obtained previously. This is the human ELOVL enzyme that allows the most important production of VLCFA (up to 7.9% of TFAs) in *Y. lipolytica*. Although PERMANOVA detected significant differences between all pairwise strain comparisons (p-value < 0.05), these results should be interpreted with caution because PERMDISP indicated heterogeneous group dispersions (*p* < 0.01), meaning that some of the detected differences may arise from unequal within-group variability rather than true shifts in centroid position. Nevertheless, the PCoA visualization (Figure S15) shows that while some strains are clearly separated from yPFK, others such as HsELOVL2 and HsELOVL4, cluster in close proximity to it, which is consistent with a lack of activity on endogenous fatty acids substrates.

Concerning elongation of exogenous PUFAs (Fig. 6B**)**, the control strain is unable to elongate any of the five PUFAs (less than 2% elongation) and the exogenous PUFA elongation rates are broadly similar between the overexpression strains with or without native YlELO2. When overexpressed, YlELO1 shows a small activity on C18:3ω3 (9.4%), while YlELO2 is slightly active on C18:2ω6 (14.4%). Overexpression of HsELOVL4 did not result in any change in profile or elongation of exogenous PUFAs. Exogenous PUFAs were not significantly elongated neither with HsELOVL1 nor with HsELOVL3. Interestingly, it appears that HsELOVL7 is capable of elongating all exogenous PUFAs but with low activity (elongation rates ranging from 7.9 to 18.1%) (Table S9). HsELOVL6 has a low activity on C18:3ω3 (9.4% elongation) and has no activity on other exogenous PUFAs. HsELOVL5 is the most active ELO tested, being active on all PUFAs tested with variable activity: moderate for C18:2ω6 and C18:3ω3 (with elongation rate of 11.4% and 16.6% respectively, and significant on C18:3ω6, C20:4ω6, and C20:5ω3 with elongation rate ranging from 51.1% to 72.4%). The other enzyme that has significant activity on PUFAs is HsELOVL2, with elongation rate of 22.2% and 44.0% on C20:4ω6, and C20:5ω3, respectively, but little or no activity on the others, nor on SFAs/MUFAs since the profile without addition is unchanged. The expression of HsELOVL2, and to a lesser extent HsELOVL5, allows the elongation of C20 PUFAs to C24, with the product being elongated a second time (Fig. 6C). It is even possible that HsELOVL2 could extend to C26:5w3 and C26:4w6, as a peak that could match is visible on the chromatogram, but insufficiently intense to be confirmed with certainty with associated mass spectra (Figure S16).

The expression of HsELOVL4 did not alter the endogenous fatty acid profile nor result in elongation of exogenous PUFAs. We hypothesize that this enzyme is either not very active when expressed in *Y. lipolytica*, or that it acts on substrates other than those present endogenously and those tested.

Based on these results, we were able to attribute the elongation reactions they catalyze in the VLCFA biosynthesis pathway to each of the enzymes except HsELOVL4 (Fig. 6D). For all reactions, at least one enzyme could be attributed, except for the elongation of monounsaturated fatty acids beyond C20:1.

## Discussion

Fatty acid elongases are key enzymes in the synthesis of very long-chain fatty acids, as they are rate-limiting and responsible for the substrate specificity of the elongation reaction. This study presents the use of *Yarrowia lipolytica* as a robust chassis for the fine characterization of the specificity of these ELO enzymes. To this end, we have developed a fast and efficient complete workflow, from strain construction to analysis. This methodology was applied to characterize the seven human ELOVLs and the two ELOs of *Y. lipolytica* on endogenous fatty acids and five exogenous polyunsaturated substrates in a single series of experiments.

By overexpressing these enzymes, we were able to confirm the substrate specificities of the two native elongases: YlELO1 for elongation from C16 to C18, and YlELO2 for elongation up to C24 and C26 [42]. Given the specificity of YlELO2, we showed that its inactivation is necessary to overcome functional redundancy and thus reveal the specificity of the heterologous elongases on saturated and monounsaturated substrates. We also proved that YlELO2 was an essential gene because despite of our attempts and as previously shown [42], we were unable to delete it. To simplify strain construction, we developed a method for knocking out YlELO2 by knock-in the heterologous elongase to be characterized, enabling the one-step construction of *elo2∆* strains overexpressing heterologous elongases. Co-integration of the DsRed fluorescent gene allows rapid identification of clones that have integrated the recombination cassette. Rather than inactivation, a strategy to inhibit YlELO2 expression could have been considered. The CRISPRi inhibition system is an effective method for reducing targeted gene expression and has already been developed in *Yarrowia lipolytica* [44–46]. Such a strategy was employed by Wang et al. to reduce the expression of the essential ∆9-desaturase YlSCD1 to increase the supply of C18:0 precursor for nervonic acid production using dynamically regulated CRISPRi [37]. However, the proportion of oleic acid in the strain is still significant, and it remains very difficult to significantly reduce the expression of an essential gene. Replacing the YlELO2 promoter could have been considered, as recently done by Zhou et al. with the copper-repressible promoter P_CTR1_ to reduce fatty acid elongation and allow the accumulation of palmitoleic acid [47, 48]. However, such approach seems less suitable for characterizing heterologous ELOs, as residual expression could still interfere with the analysis.

So far, the limiting step in strain characterization has been the cultivation and analysis of fatty acids. Indeed, characterization on exogenous substrates requires multiple culture conditions, making it laborious to perform in flasks and using the direct transmethylation method in glass tubes [41]. It is the reason why we have developed a protocol to perform all these steps in a 96-well format, significantly increasing the screening throughput. Thanks to miniaturization, we were able to cultivate and analyze the fatty acid content of more than 312 culture conditions (four replicates of 13 strains in six media, regardless replicates between different plates) in a single batch, all in less than two weeks.

*Yarrowia lipolytica*’s culture miniaturization can be challenging as it is a demanding species (such as for its strictly aerobic metabolism or its predisposition to filamentous growth), its cultivation in small volume can lead to perturbations of its phenotype that can induce a lack of reproducibility [49]. Herein, we set up growth conditions in 96-well deepwell plates that allows to reach similar cell densities and comparable fatty acid profiles than those obtained in flasks. Comparable culture conditions were used in engineering work on *Y. lipolytica* to screen a library of mutants for erythritol production or to improve the assimilation of methanol, a non-native substrate [50, 51].

While the culture conditions could be successfully adapted to a small format in different organisms for fatty acid production, we report herein for the first time, a method for direct transmethylation and fatty acid extraction in a 96-well format, significantly increasing the analysis throughput. Direct transmethylation in acidic methanol without prior lipid extraction is a widely used protocol for the analysis of total fatty acids in lipid-rich organisms such as microalgae [52, 53], thraustochyrids [54, 55] or fungi [56], but also in plasma [57] or plant tissues [58, 59]. This procedure requires incubation of the freeze-dried biomass in acidic methanol at a very high temperature (80 °C or higher) for several hours. These conditions require perfectly sealed vessels, typically glass tubes with screw caps, to prevent methanol evaporation, which is difficult to achieve in a deepwell, despite thermal sealing. To overcome evaporation, the temperature was reduced below the boiling point of methanol (64.7 °C), but the reaction duration was increased to 24 h (versus 4 h) to obtain an adequate reaction yield. The fatty acid concentrations obtained are generally like those obtained with the standard protocol in glass tubes, with the exception of very long-chain saturated fatty acids, which appear to be less well extracted. Despite several attempts, we were unable to significantly increase their recovery. As the solubility of saturated VLCFAs in methanol appears to be the limiting factor, addition of a co-solvent such as dichloromethane could overcome this limitation. This strategy was not implemented in the present work but represents a relevant option for future optimization. Despite this limitation, the miniaturized method remains robust for elongase specificity screening with an increased throughput. For applications requiring exhaustive VLCFA quantification, it can be complemented with standard extraction and transmethylation procedures. To our knowledge, no high-throughput protocol has yet been developed based on this widely used method, although a method using TMSH has been developed for high-throughput analysis of *Chlamydomonas reinhardtii* [60]. This method has the advantage that it can be performed in a single step at room temperature. Adapting this protocol to a 96-well format can also be considered.

Using our full workflow, we were able to characterize the seven human ELOVLs in a single series of experiments on endogenous saturated and monounsaturated substrates, as well as on the six mains exogenous PUFAs. HsELOVL1, HsELOVL3 elongates saturated and monounsaturated endogenous fatty acids in *Y. lipolytica* and are thus able to complement YlELO2 activity, but they do not show any activity on PUFA. On the other hand, HsELOVL2, HsELOVL5, and to a lower extent HsELOVL7 are active on polyunsaturated substrates only and are neither able to modify the fatty acid profile on endogenous fatty acid of *Y. lipolytica* nor to complement the YlELO2 activity. HsELOVL7 has a wide fatty acid specificity as it is both able to elongate saturated, monounsaturated and polyunsaturated substrate efficiently. We were thus able to assign the elongation reactions catalyzed by each enzyme, with the exception however of HsELOVL4. Even if we cannot exclude a poor expression of this enzyme in a yeast expression system, it is more likely that its own substrates are not present endogenously in *Y. lipolytica.* Indeed, HsELOVL4 is involved in the elongation of fatty acids with more than 26 carbons [61, 62]. However, the exogenous addition of such fatty acids is difficult, since saturated fatty acids are poorly soluble, even in emulsion form, and the cost of such unsaturated fatty acids is high. For all other enzymes, the results of characterizations are similar to those found in the literature on these enzymes, which have been studied for over twenty years, demonstrating the robustness of our workflow [17, 18, 62, 63].

None of the human elongases tested was able to produce monounsaturated fatty acids longer than C20:1, even though they are naturally synthesized in humans, particularly nervonic acid (C24:1∆15) which is abundant in the human brain, where it accumulates to form sphingomyelin [64]. Studies have shown that HsELOVL1 is involved in the elongation of monounsaturated fatty acids from C20:1 to C26:1. However, the fraction of endogenous C20:1 in our chassis is probably too small to have any elongation activity, so it would be necessary to add it exogenously to complete the characterization.

In mammals, there are two pathways for the synthesis of C20-PUFAs: (1) the ∆6 pathway: ∆6-desaturation, then ∆6-elongation; (2) the ∆9 pathway: ∆9-elongation, then ∆8-desaturation [65]; the key enzymes in both pathways being HsELOVL5 elongase and the FADS2 desaturase (Figure S17). Nevertheless, the ∆6 pathway is the main route, favored by higher activity of the desaturase as well as elongase on substrates of this pathway [66, 67]. Our results measuring conversion rates in yeast confirm these observations for the elongases. The activity of HsELOVL5 and HsELOVL7 on ∆9 substrates shows low conversion rates (11.1 to 17.7%), while HsELOVL5 shows high activity (72.4% conversion rate) on ∆6 substrate (C18:3ω6). These results may explain, at least partly, why the ∆6 pathway is the main pathway in mammals, but it should be kept in mind that the level of activity of these enzymes depends on much more complex and finely regulated mechanisms, such as the expression levels of different ELOs or post-translational modifications.

## Conclusion

In this work, we established an improved methodology to characterize in vivo fatty acid elongases in *Yarrowia lipolytica*. A strain engineering strategy was developed to integrate elongase expressing cassettes into the genome by CRISPR-Cas9 mediated homologous recombination, using the fluorescent reporter gene DsRed to easily select clones having integrated the construct. To increase characterization throughput, cultures and fatty acid analysis were miniaturized to 96-well format. As a demonstration, we characterized in the same series of experiments the seven human ELOVLs and the two native ELOs from *Y. lipolytica* on endogenous saturated and monounsaturated fatty acids and on five exogenous PUFAs. Simply by overexpressing an elongase, with or without deletion of the native YlELO2, we were able to produce a variety of fatty acid modifications, demonstrating the importance of selecting elongases with the adequate specificity to achieve the synthesis of VLCFAs of interest. The workflow disclosed herein enables fast and easy screen of fatty acid-active enzymes, such as elongases or desaturases, thus opening new insight to diversify and produce a large array of fatty acids in *Yarrowia lipolytica*.

## Supplementary Information

Supplementary Material 1.

Supplementary Material 2.

## Data Availability

All data generated or analyzed during this study are included in this published article and its supplementary information files.

## Abbreviations

ELO/ELOVL: Fatty acid elongase
FAME: Fatty acid methyl ester
GC-FID/MS: Gas chromatography with flame ionization detector and coupled with mass spectrometry
gRNA: Guide RNA
HR: Homologous recombination
MUFA: Monounsaturated fatty acid
NAT: Nourseothricin
NatR: Nourseothricin N-acetyl transferase
NHEJ: Non-homologous end-joining
PCoA: Principal Coordinates Analysis
PUFA: Polyunsaturated fatty acid
SFA: Saturated fatty acid
TFA: Total fatty acids
VLCFA: Very-long chain fatty acid
YPD: Yeast peptone dextrose medium
YNB: Yeast nitrogen base medium
