# Supplementary material for: A workflow to explore elongase diversity and extend the repertoire of fatty acids produced by Yarrowia lipolytica
Source: Microb Cell Fact. 2025 Dec 23;25:18. doi: 10.1186/s12934-025-02890-y (PMC12837234; doi:10.1186/s12934-025-02890-y)
Supplement: Supplementary file 2 — Supplementary Material 2. [file 12934_2025_2890_MOESM2_ESM.docx]

# Supplementary Figures

**A workflow to explore elongase diversity and extend the repertoire of fatty acids produced by Yarrowia lipolytica**

Jérémy Le Reun^1^, Zélie Salvioli^1^, Christian Croux^1^, Jérémy Esque^1^, Isabelle André^1,*^ and Florence Bordes^1,*^

(1) Toulouse Biotechnology Institute, TBI, Université de Toulouse, CNRS, INRAE, INSA, Toulouse, France

(*) corresponding authors: [isabelle.andre@insa-toulouse.fr](mailto:isabelle.andre@insa-toulouse.fr); [bordes@insa-toulouse.fr](mailto:bordes@insa-toulouse.fr)

- Figure S1 : pCg71 plasmids used to express CRISPR-Cas9 system components, including one gRNA expression cassette.
- Figure S2 : pCg72 plasmids used to express CRISPR-Cas9 system components, including two gRNA expression cassettes.
- Figure S3 : pHR plasmids carrying recombination templates for targeted gene insertion in Y*. lipolytica* genome.
- Figure S4 : Ordination of fatty acid profiles from yPFK strains overexpressing native elongases.
- Figure S5 : Ordination of fatty acid profiles from yPFK strains overexpressing human ELOVL7 (HsELOVL7).
- Figure S6 : Estimation of guide cutting efficiency for RNA guide targeting YlELO2 (YALI1_B26350g).
- Figure S7 : Colonies with the DsRed-ELO cassette integrated are easily identified by fluorescence on transformation plates.
- Figure S8 : Clones resulting from transformation transferred to a new plate still keep the fluorescence.
- Figure S9 : Non-fluorescent clones from yPFK transformation are non-edited at cut site in D1 locus.
- Figure S10 : Non-fluorescent clones from yPFK transformation are non-edited at cut site in ELO2 locus.
- Figure S11 : Fatty acid profiles are similar between yPF and yPFK strains.
- Figure S12 : Ordination of fatty acid profiles of yPFK strains expressing elongases to compare the standard (Std) and miniaturized (Mini) fatty acid transmethylation procedures.
- Figure S13 : The fatty acid profiles obtained are similar between the cultivation methods.
- Figure S14 : Fatty acid profiles and concentrations from the miniaturized workflow are similar across cultures and replicates
- Figure S15 : Ordination of fatty acid profiles of yPFK strains expressing the seven human elongases cultivated without exogenous fatty acid addition and analyzed using the newly developed miniaturized method.
- Figure S16 : HsELOVL2 and HsELOVL5 are able to elongates C20:4ω6 and C20:5ω3 for two elongation cycles.
- Figure S17 : ∆6 elongation pathway (circled in blue dashed line) is preferred over the ∆9 pathway to produce PUFAs in humans.


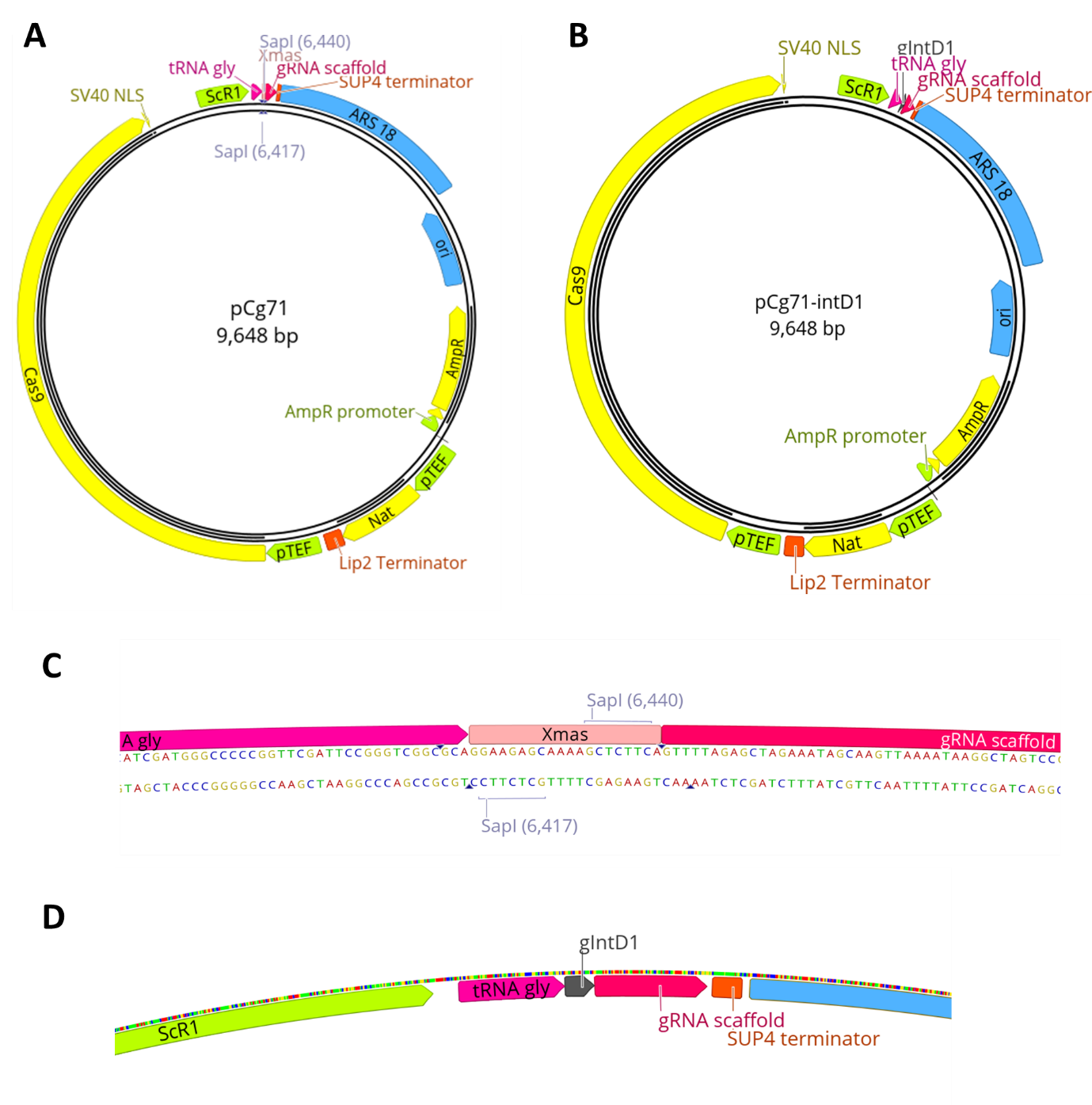


**Figure S1 : pCg71 plasmids used to express CRISPR-Cas9 system components, including one gRNA expression cassette.** These plasmids and methods for gRNA sequence insertion are adapted from Borsenberger et al. [1,2]. (A) Empty pCg71. (B) pCg71 carrying gRNA sequence to target D1 locus. (C) Site for gRNA sequence insertion using SapI type IIS enzyme. (D) gRNA expression cassette in pCg71-int D1 plasmid.


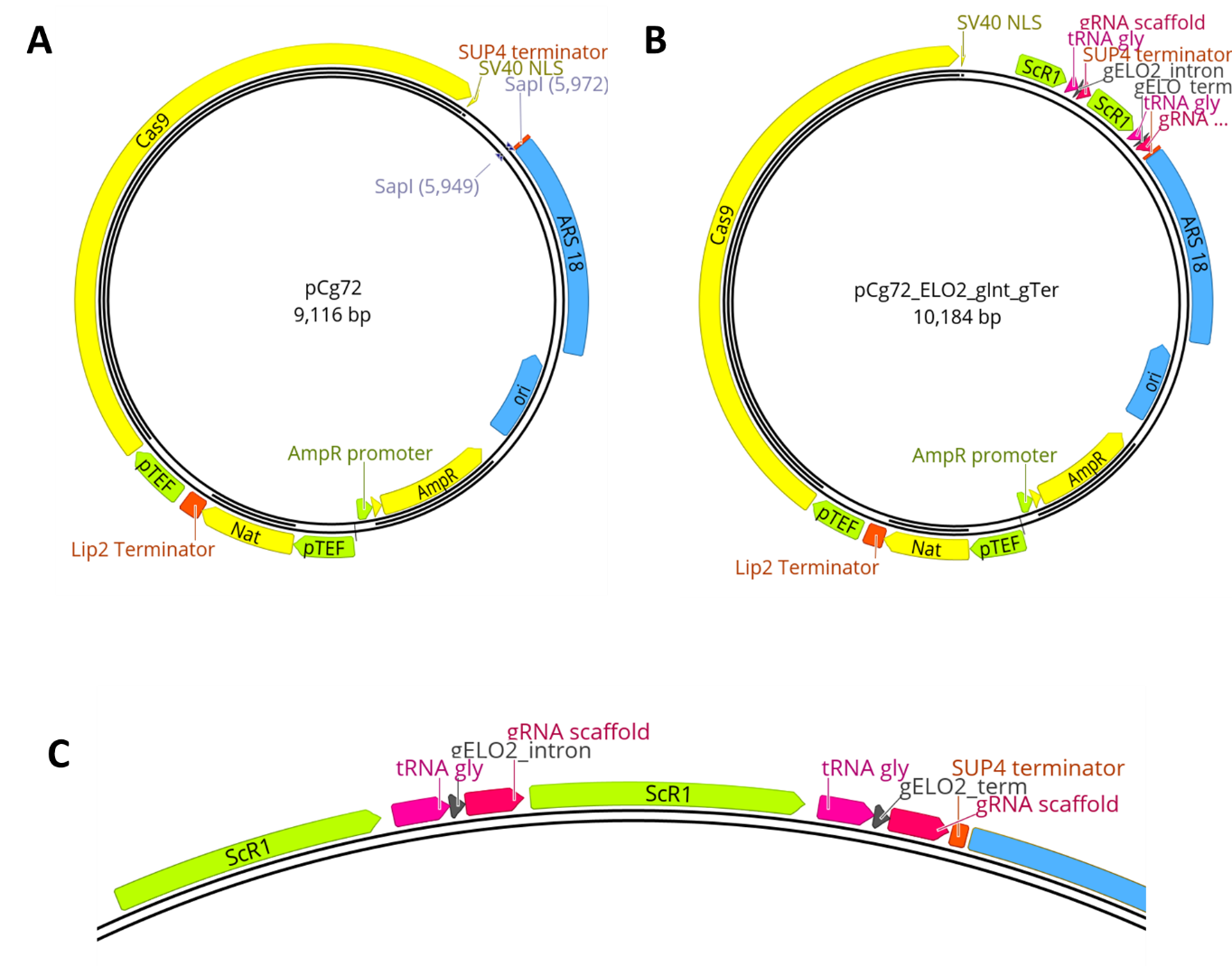


**Figure S2 : pCg72 plasmids used to express CRISPR-Cas9 system components, including two gRNA expression cassettes.** These plasmids and methods for gRNA sequence insertion are adapted from Borsenberger et al. [1,2]. (A) Empty pCg72. (B) pCg71 carrying two gRNA expression cassettes, here targeting ELO2 gene. (C) Sequence of the two gRNA expression cassettes in pCg72-ELO2_gInt-gTer.


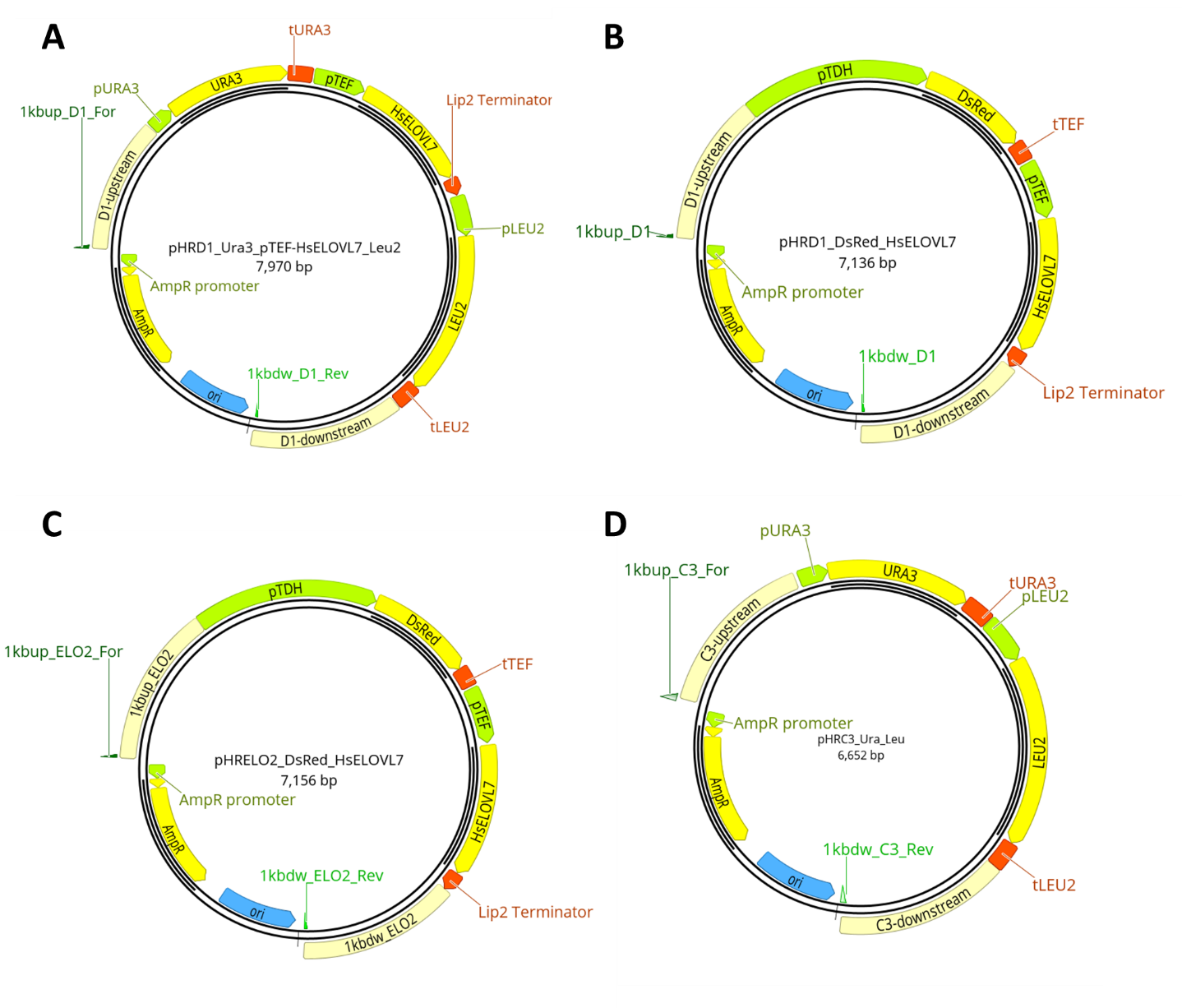


**Figure S3 : pHR plasmids carrying recombination templates for targeted gene insertion in Y. lipolytica genome.** Recombination templates are linearized by PCR amplification from 1kbup_XX_For and 1kbdw_XX_Rev primers. (A) pHRD1_Ura3_pTEF-HsELOVL7_Leu2 for insertion of HsELOVL7 expression cassette together with Ura3 and Leu2 auxotrophic markers into neutral D1 locus. (B) pHRD1_DsRed-HsELOVL7 for insertion of HsELOVL7 expression cassette and the fluorescence marker DsRed into neutral D1 locus. (C) pHRELO2_DsRed-HsELOVL7 for insertion of HsELOVL7 expression cassette and the fluorescence marker DsRed into ELO2 locus (knock-out by knock-in). (D) pHRC3_Ura3_Leu2 plasmid used to integrate Leu2 and Ura3 auxotrophic markers in neutral C3 locus. Used to make yPF^--^ strain prototrophic.

**A**


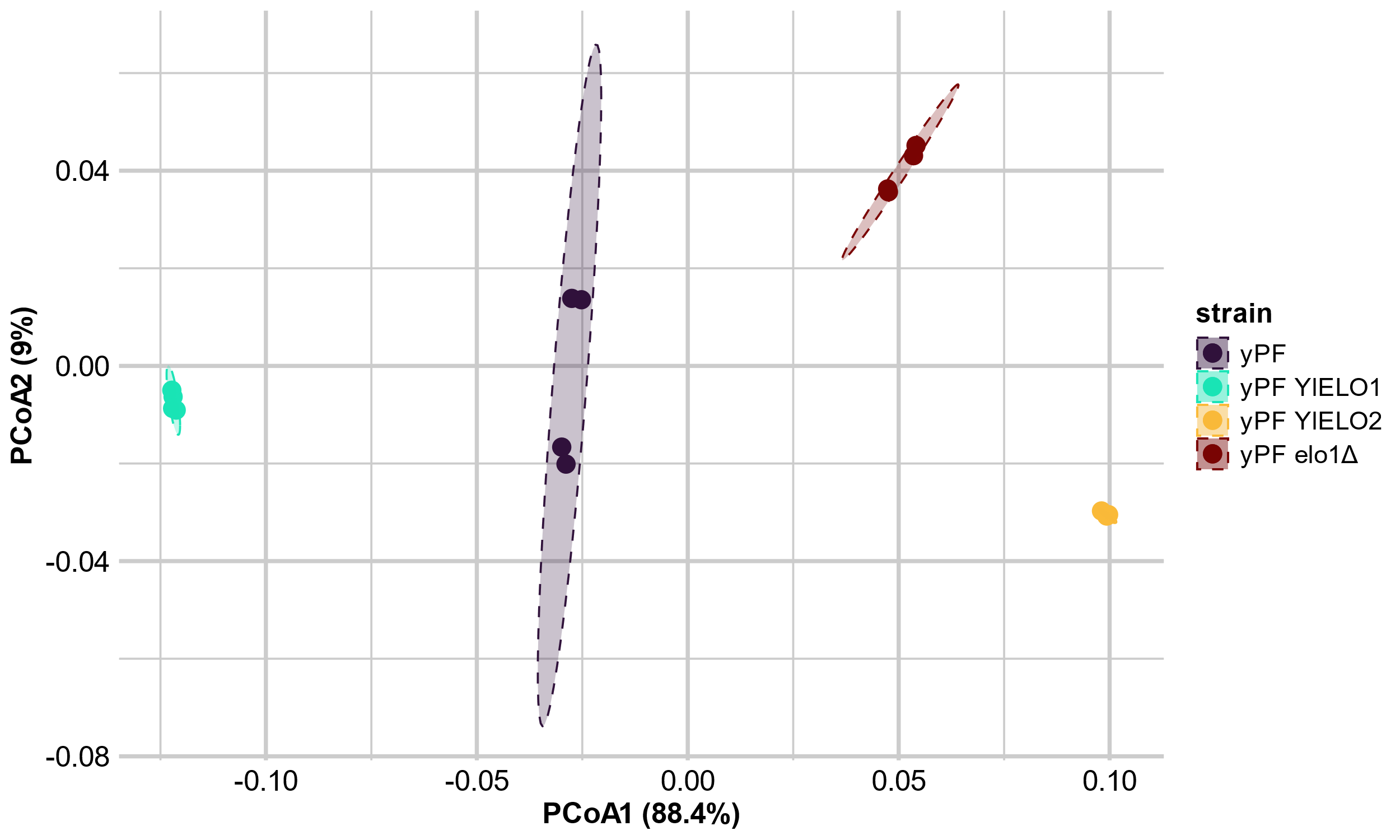
**B**


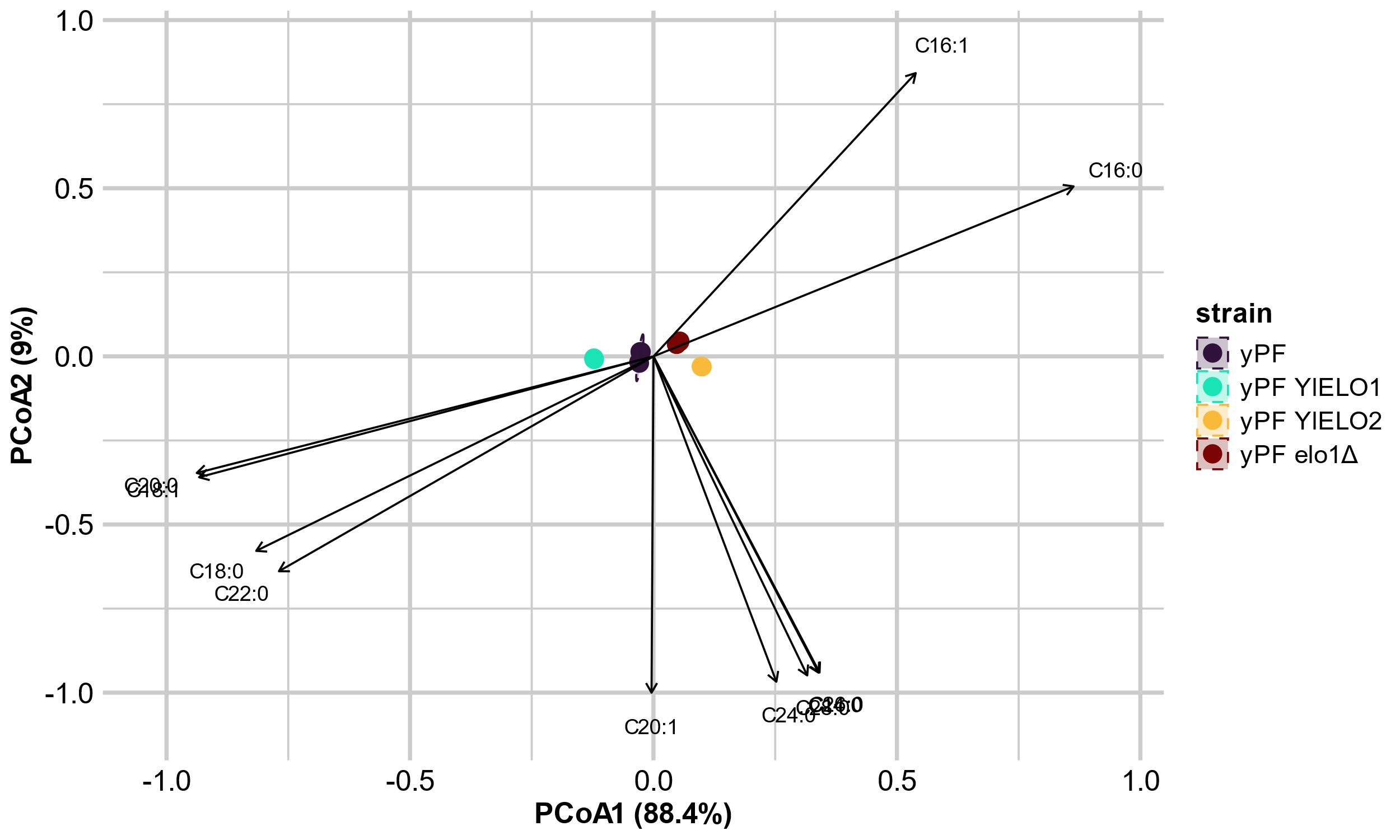
Figure S**4** : **Ordination of fatty acid profiles from yPFK strains overexpressing native elongases.** (A) Principal Coordinates Analysis (PCoA) based on Bray-Curtis distances of Hellinger-transformed fatty acid profiles. Points represent individual samples and dashed ellipses indicate 95% confidence intervals for each strain. (B) PCoA with overlaid envfit vectors for individual fatty acids. Arrows indicate the direction and relative contribution of each fatty acid to the observed separation among strains. The separation of points is identical to panel A, but arrows illustrate the variables driving strain differentiation.

**A**


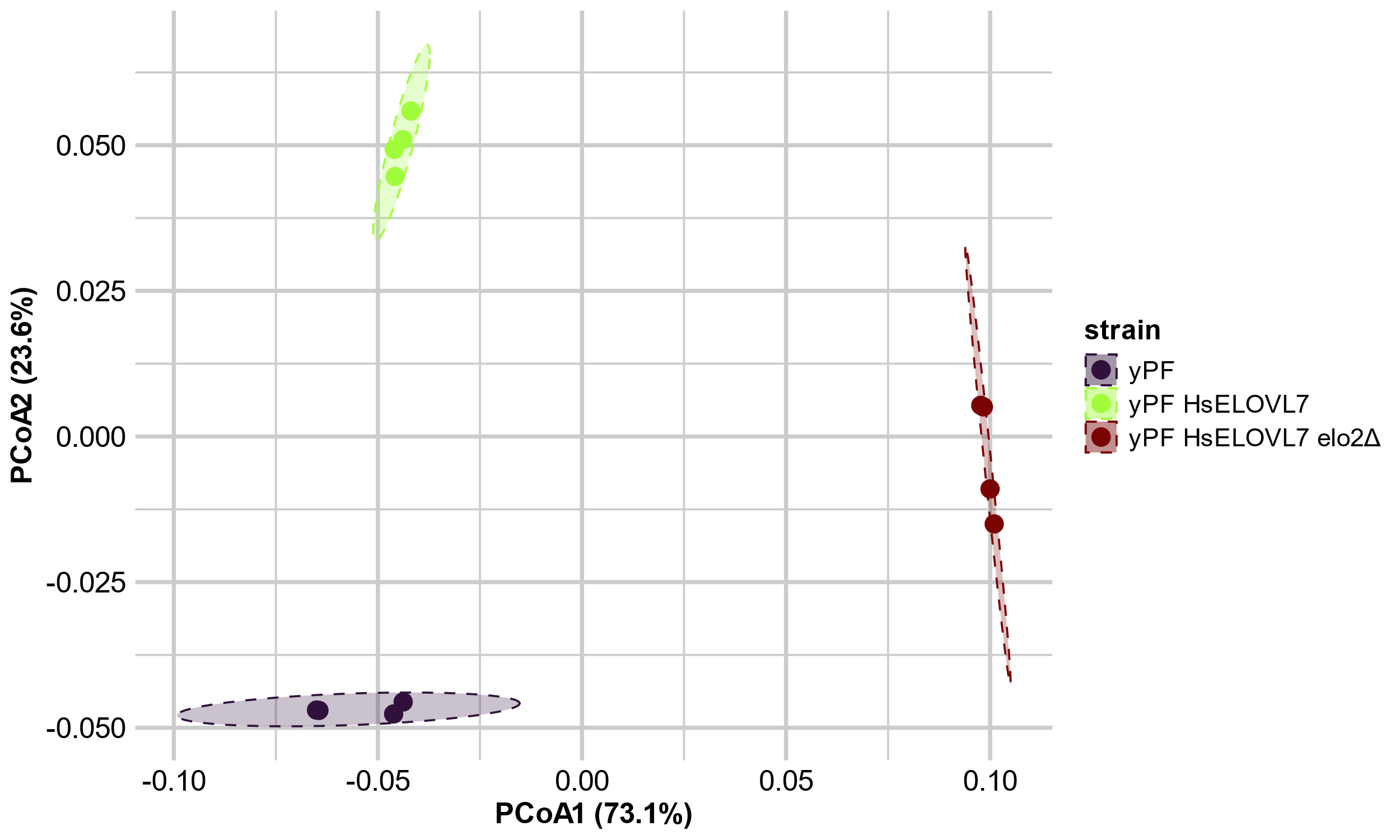
 **B**
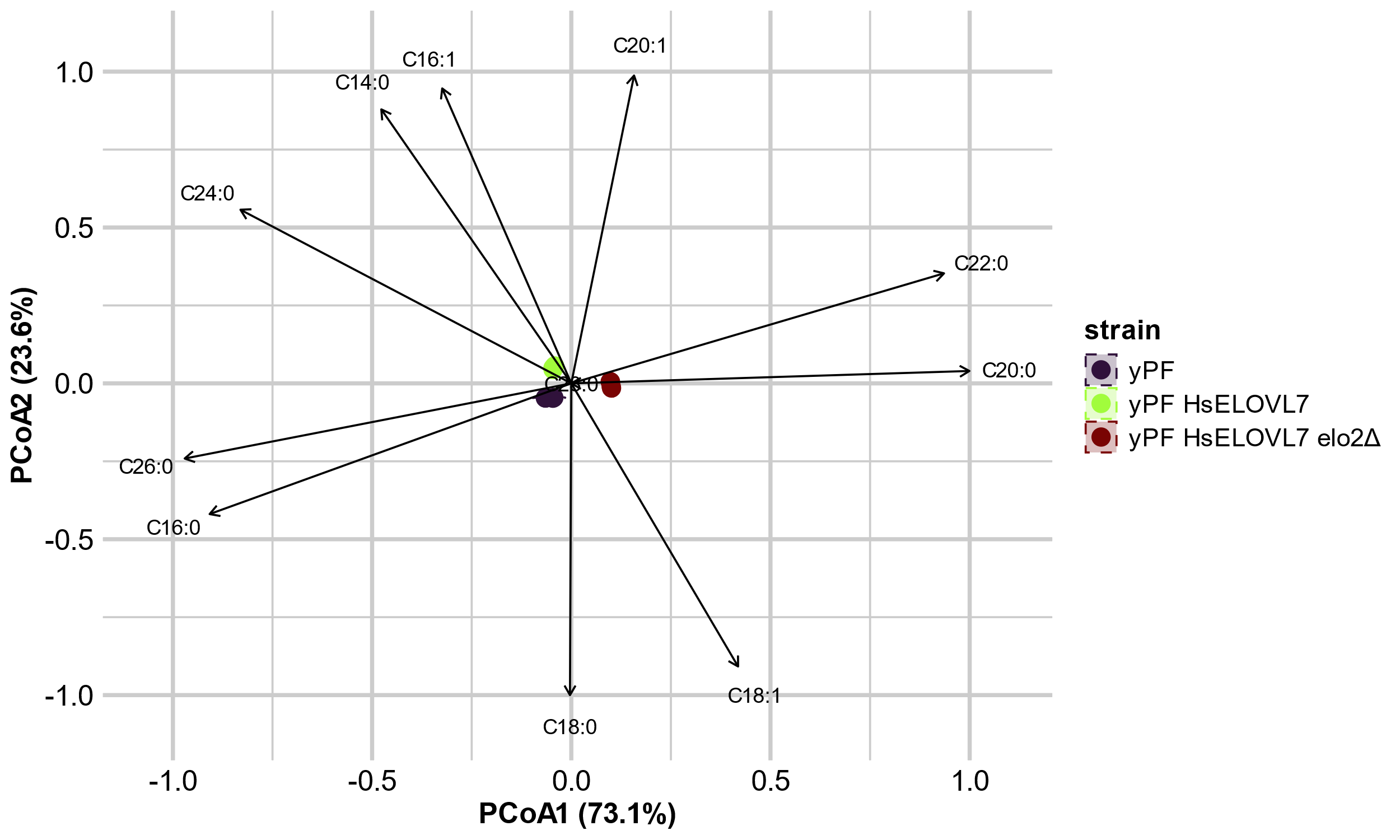


**Figure S5 :** **Ordination of fatty acid profiles from yPFK strains overexpressing human ELOVL7 (HsELOVL7).** **(A)** Principal Coordinates Analysis (PCoA) based on Bray-Curtis distances of Hellinger-transformed fatty acid profiles. Points represent individual samples and dashed ellipses indicate 95% confidence intervals for each strain. **(B)** PCoA with overlaid envfit vectors for individual fatty acids. Arrows indicate the direction and relative contribution of each fatty acid to the observed separation among strains. The separation of points is identical to panel A, but arrows illustrate the variables driving strain differentiation.


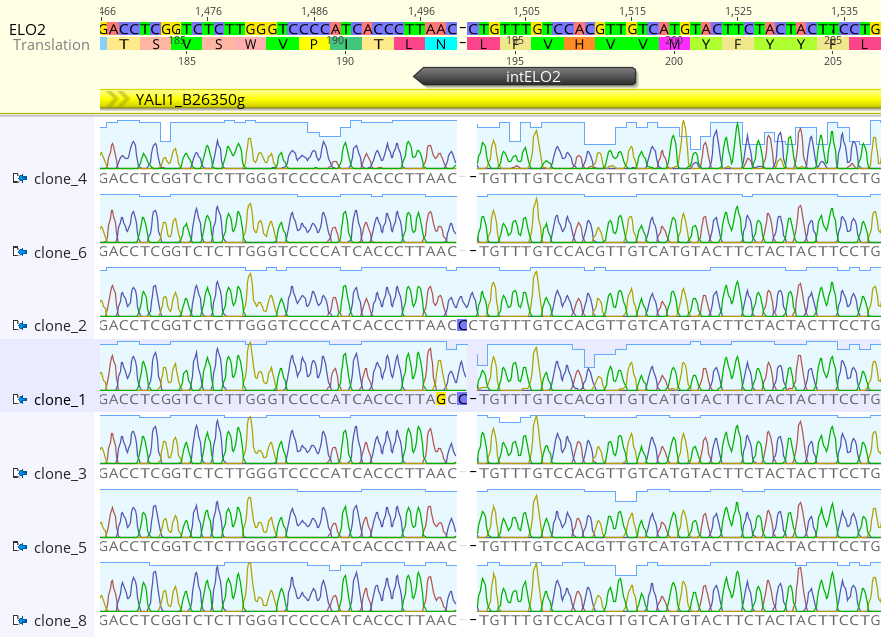


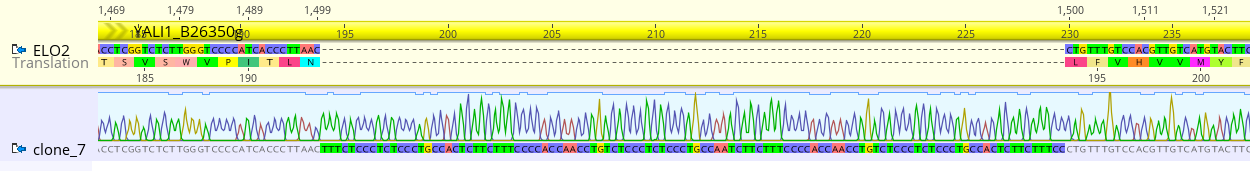


**Figure S6 : Estimation of guide cutting efficiency for RNA guide targeting YlELO2 (YALI1_B26350g).** Chromatograms obtained for 8 colonies of yPF-HsELOVL7 transformed with pCg71-intELO2 alone. For clarity purpose due to large insertion, clone 7 chromatogram, in which a large insertion occurred at cut site, was represented separately. Sequence in yellow on the top represents the genomic sequence. Guide RNA used to target native gene, gIntELO2, is represented in black.


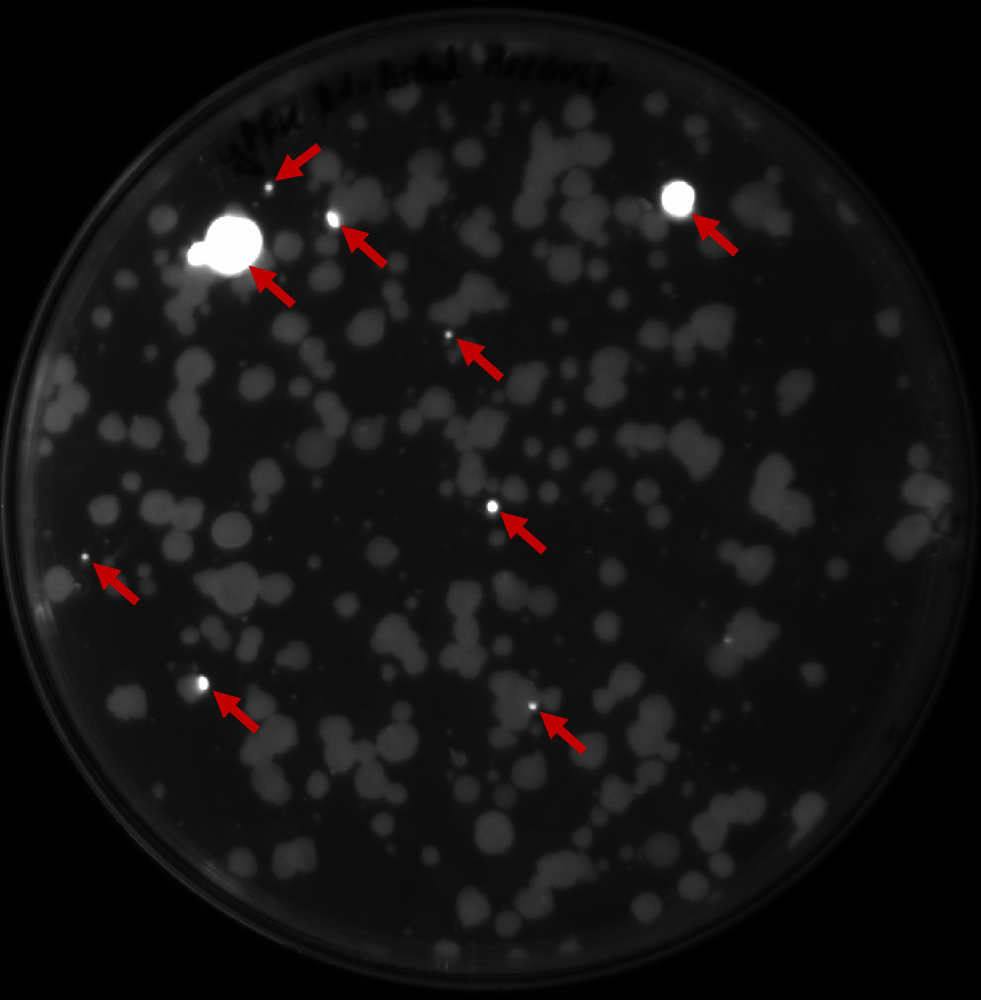


**Figure S7 : Colonies with the DsRed-ELO cassette integrated are easily identified by fluorescence on transformation plates.** Enlargement of transformation plate for D1::DsRed-HsELOVL7 transformation in yPFK strain represented in the main text Figure 4. Red arrows highlight fluorescent colonies.


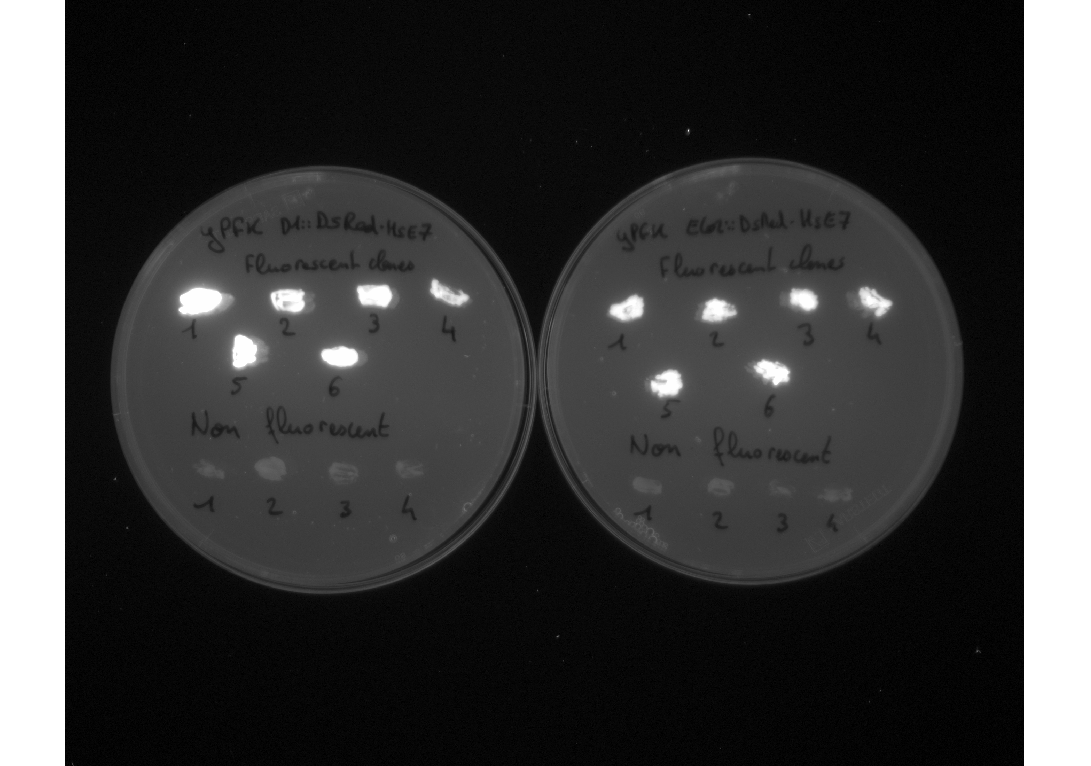


**Figure S8 : Clones resulting from transformation transferred to a new plate keep the fluorescence.** Individual colonies from transformation plates for DsRed-HsELOVL7 transformation in either D1 (left plate) or ELO2 (right plate) locus were transferred to a new plate and visualized for fluorescence.
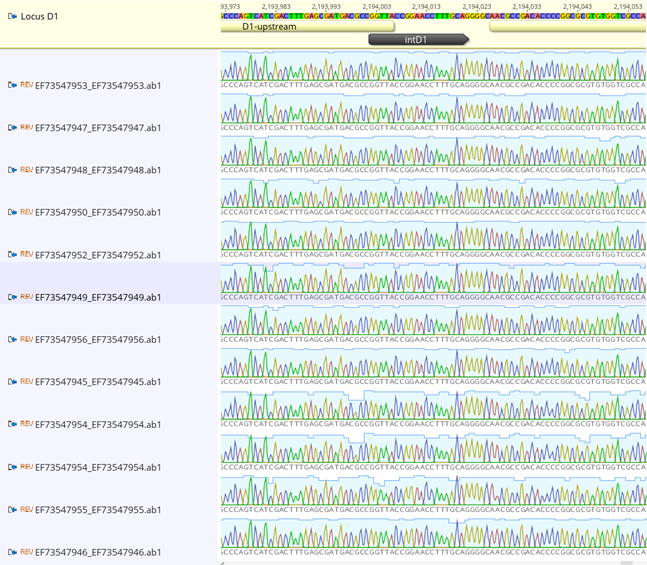


**Figure S9 : Non-fluorescent clones from yPFK transformation are non-edited at cut site in D1 locus.** Sequencing of D1 locus in 11 non-fluorescent yPFK clones transformed for D1: DsRed-HsELOVL7 insertion using intD1 guide. Sequence in yellow on the top represents the genomic sequence. Guide RNA used to target locus, gIntD1, is represented in black.

**
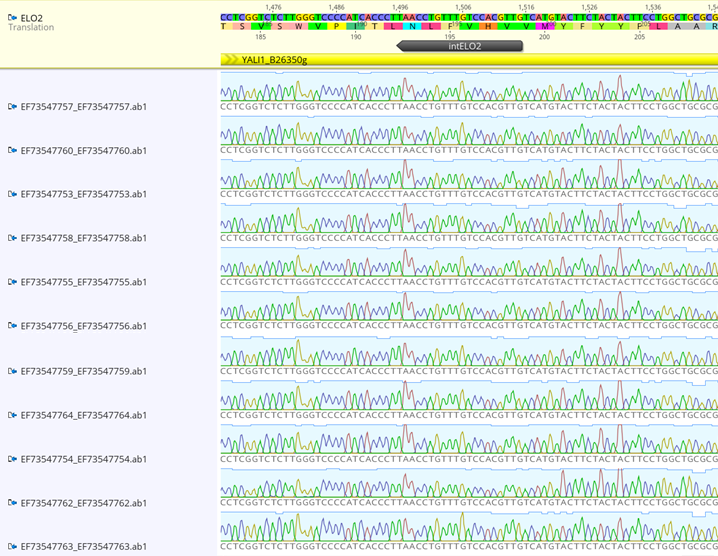
**

**Figure S10 : Non-fluorescent clones from yPFK transformation are non-edited at cut site in ELO2 locus.**  Sequencing of ELO2 locus in 11 non-fluorescent yPFK clones transformed for ELO2: DsRed-HsELOVL7 insertion using intELO2 guide. Sequence in yellow on the top represents the genomic sequence. Guide RNA used to target locus, IntELO2, is represented in black.

**Figure S11 : Fatty acid profiles are similar between yPF and yPFK strains.**  Comparison of fatty acid profiles obtained in flasks between strains overexpressing ELO constructed using the two integration methods. For yPF strains (pox1-6Δ, fad2Δ), ELO integration is performed at the D1 locus with secondary selection by auxotrophy, followed or not by deletion of the native gene by CRISPR-Cas9. For yPFK strains (pox1-6∆, fad2∆, ku70∆), ELO and the DsRed expression cassette are integrated either at the D1 locus or into the native ELO2 gene for its inactivation. Cultures were grown in triplicate in flasks containing 15 mL of YT2D5 for 48 hours. Fatty acids were extracted and quantified using the standard method.

**A**

**
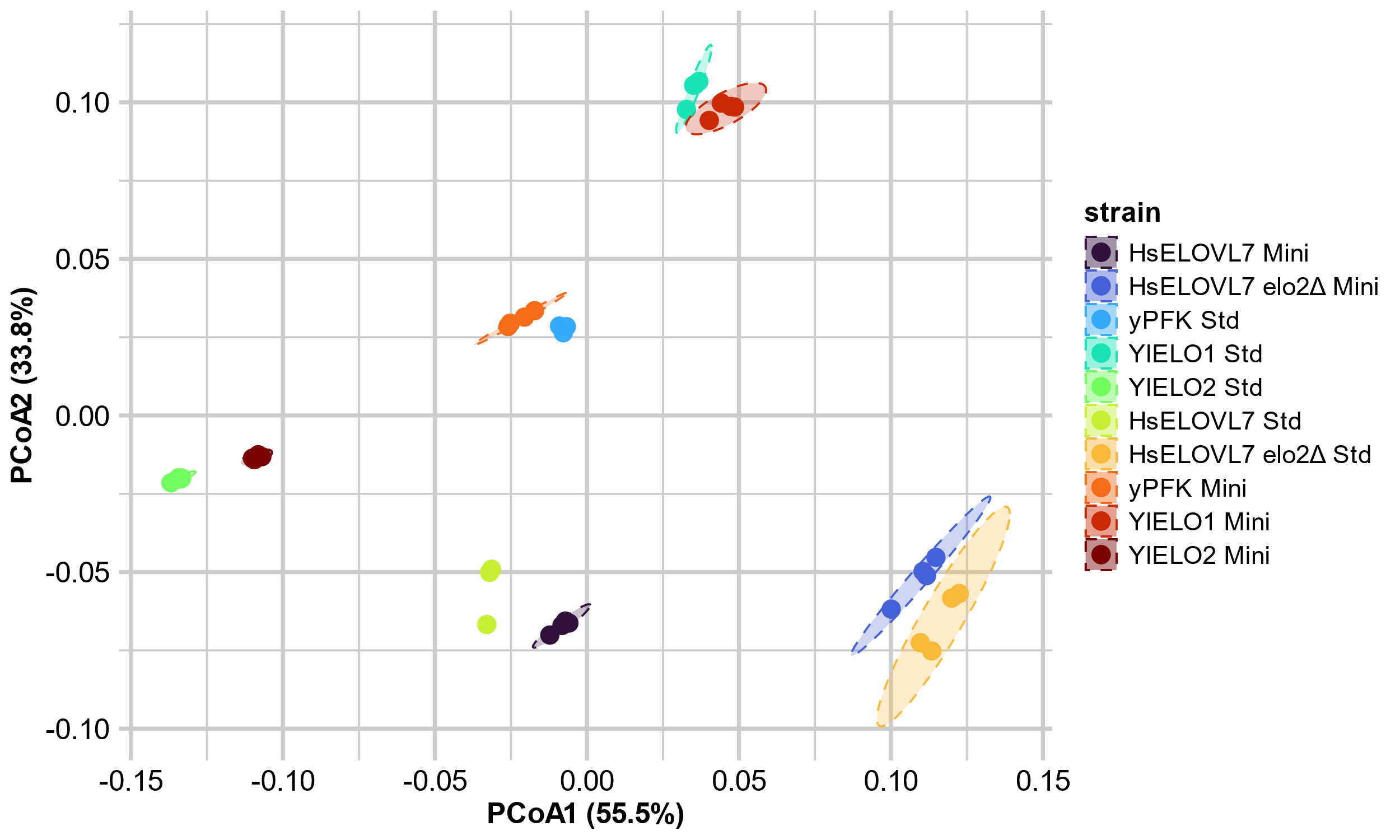
**

**B**

**
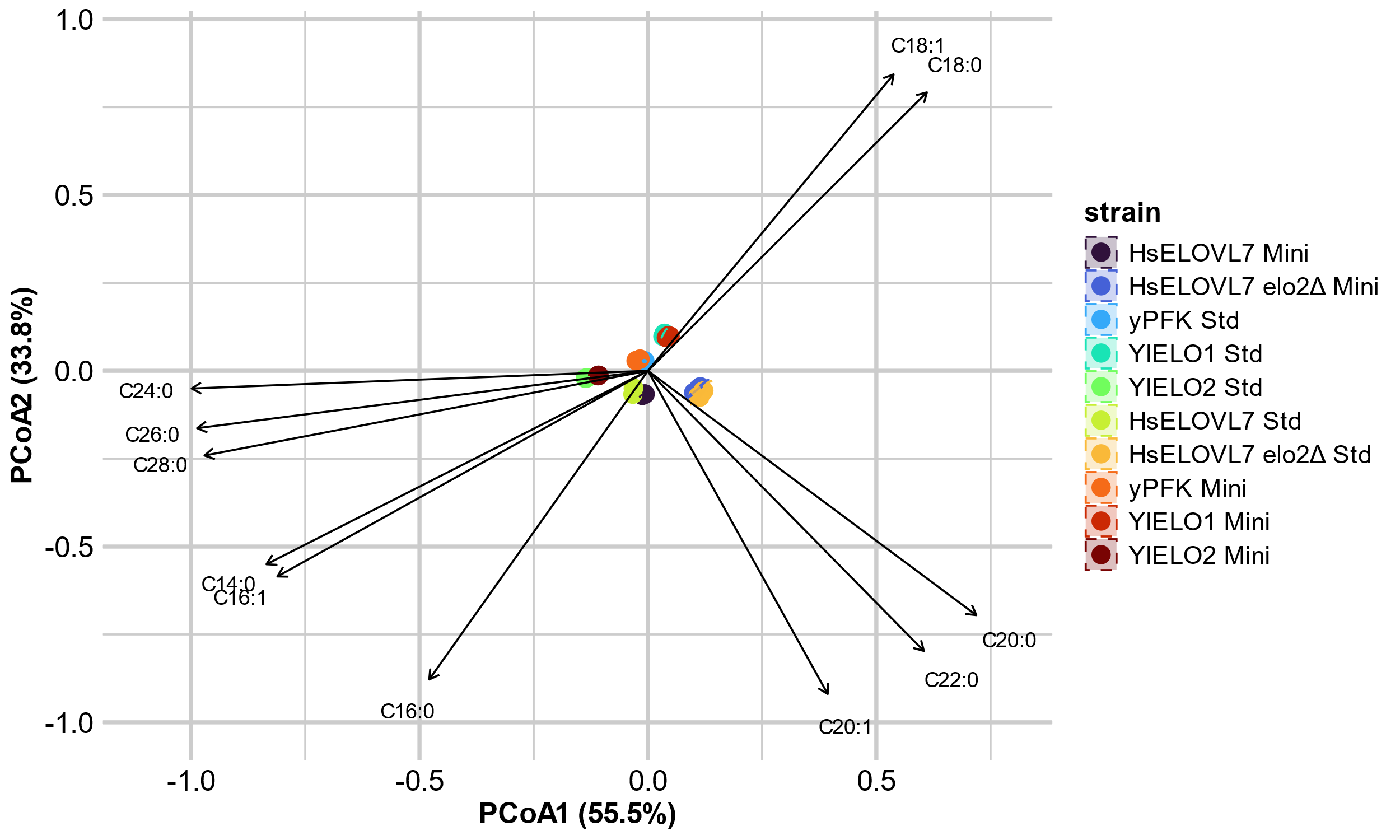
**

**Figure S12 : Ordination of fatty acid profiles of yPFK strains expressing elongases to compare the standard (Std) and miniaturized (Mini) fatty acid transmethylation procedures.** (A) Principal Coordinates Analysis (PCoA) based on Bray-Curtis distances of Hellinger-transformed fatty acid profiles. Points represent individual samples and dashed ellipses indicate 95% confidence intervals for each strain. Due to colinear datapoints and small number of replicates (3), ellipse couldn’t be drawn for HsELOVL7 Std. (B) PCoA with overlaid envfit vectors for individual fatty acids. Arrows indicate the direction and relative contribution of each fatty acid to the observed separation among strains. The separation of points is identical to panel A, but arrows illustrate the variables driving strain differentiation.

**A**

**
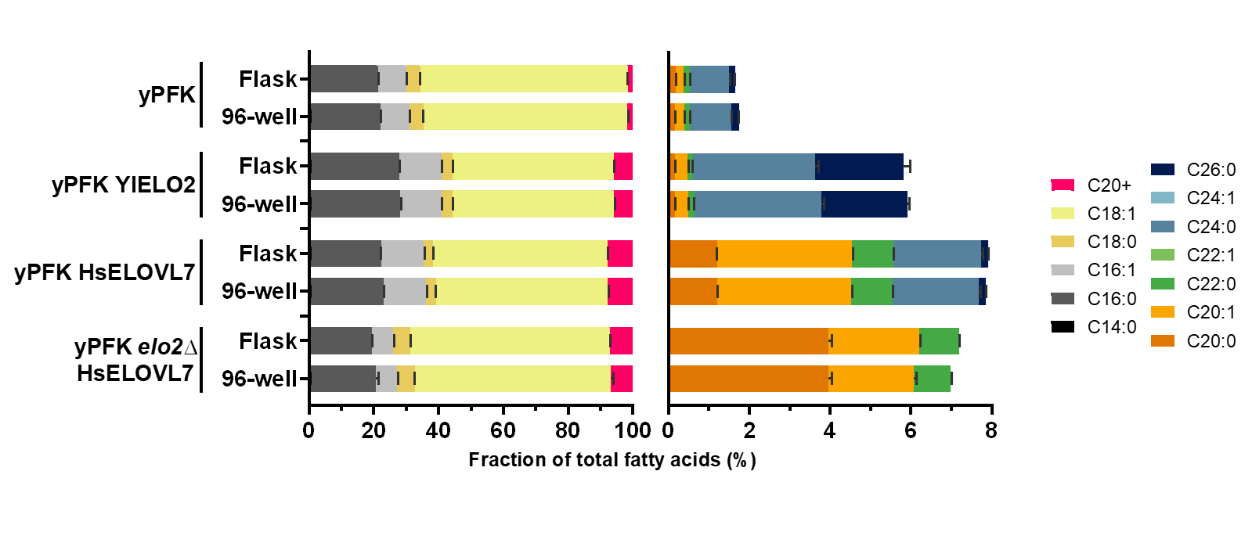
**

**B**
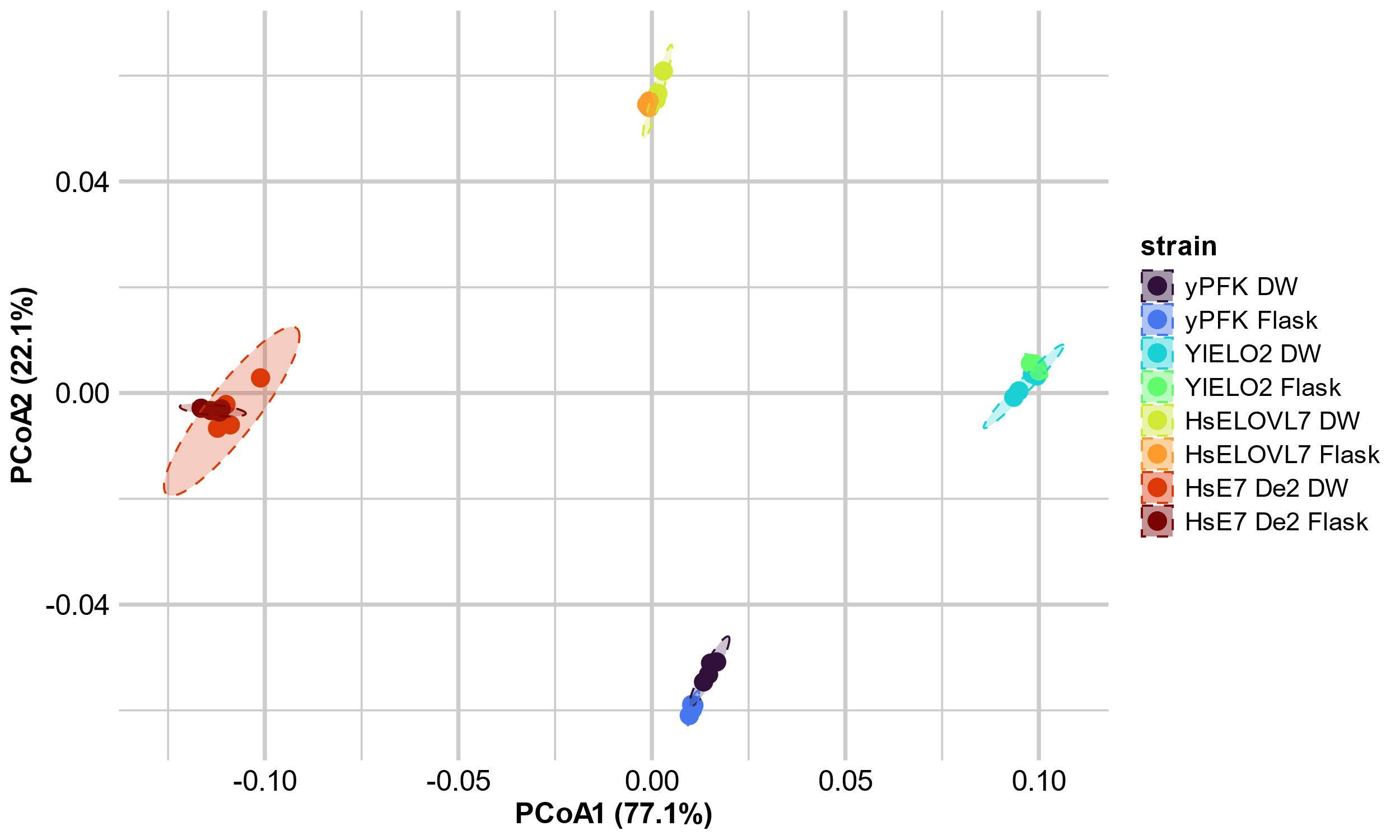


**Figure S13 : The fatty acid profiles obtained are similar between the cultivation methods.** Fatty acid profiles of yPFK strains expressing elongases cultivated in flasks or in 96-well plate (DW) and analyzed using miniaturized fatty acid transmethylation procedure were compared. **(A)** Fatty acid content of the strains was analyzed by GC-FID/MS. The data represent the mean ± standard deviation (n = 4). (A) Fatty acid profiles represented as fractions of total fatty acids (TFA). Left panel shows the long chain fatty acids (C14-C18), while the right panel shows an enlargement of the VLCFA (C20+) fraction. (B) Principal Coordinates Analysis (PCoA) based on Bray-Curtis distances of Hellinger-transformed fatty acid profiles. Points represent individual samples and dashed ellipses indicate 95% confidence intervals for each strain.


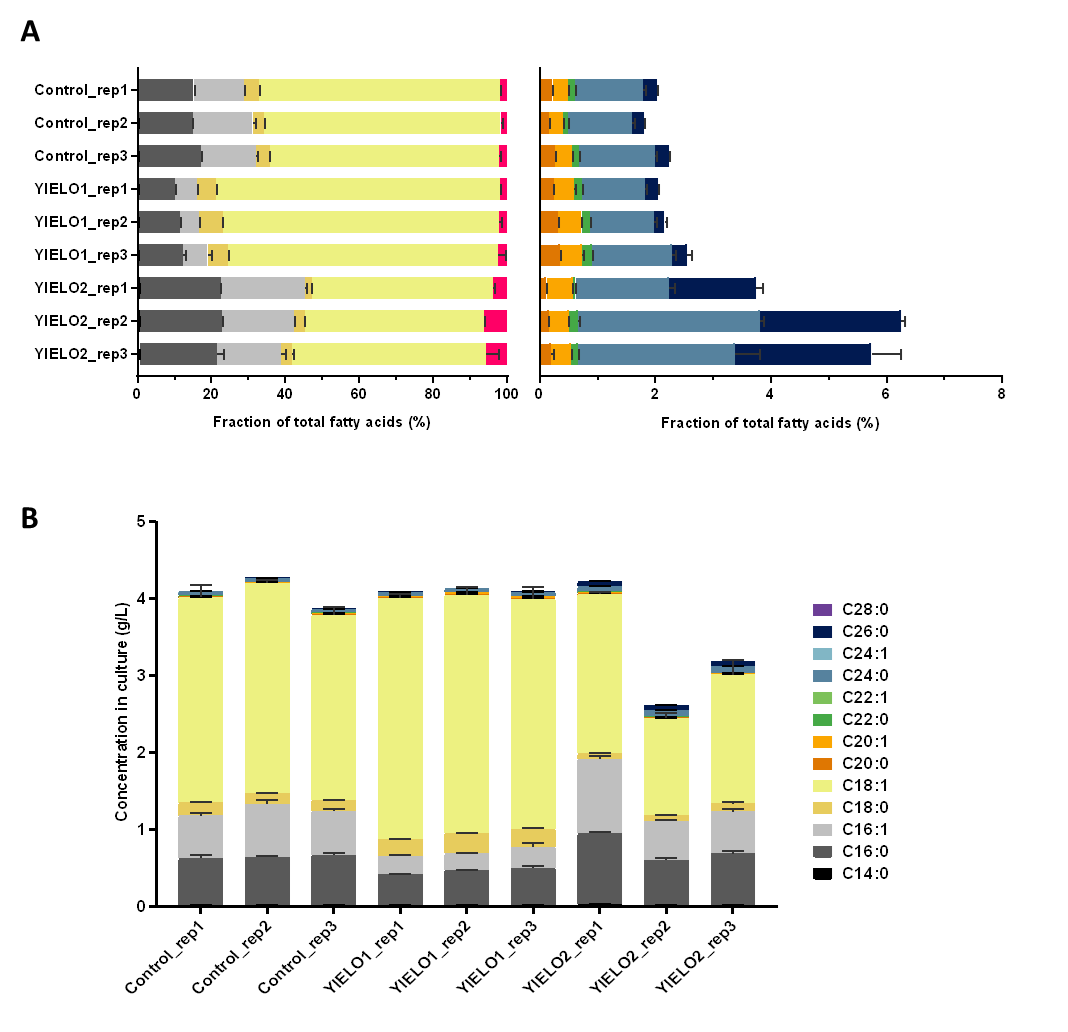


**Figure S14 : Fatty acid profiles and concentrations from the miniaturized workflow are similar across cultures and replicates. Comparison of fatty acid concentrations obtained**

**A
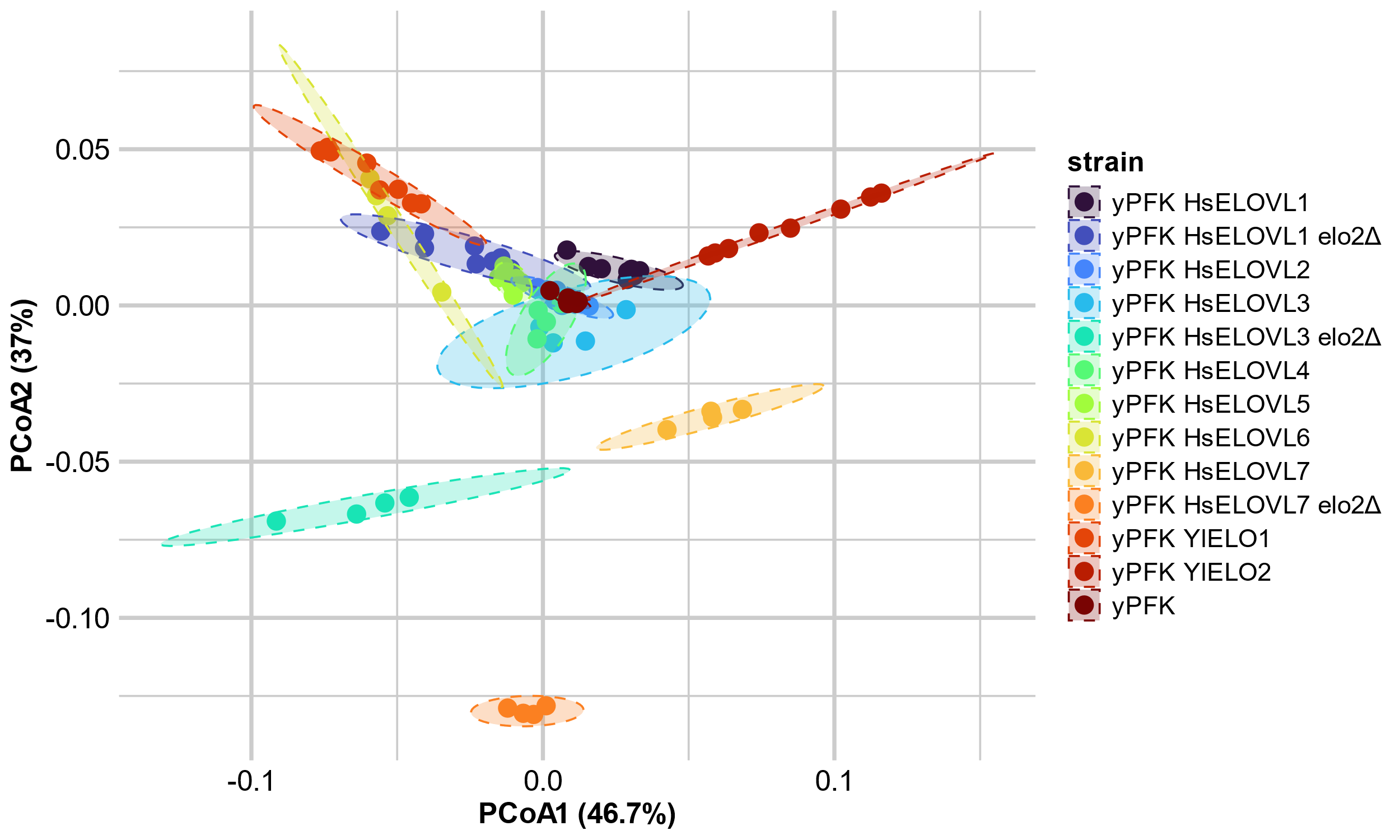
**

**B
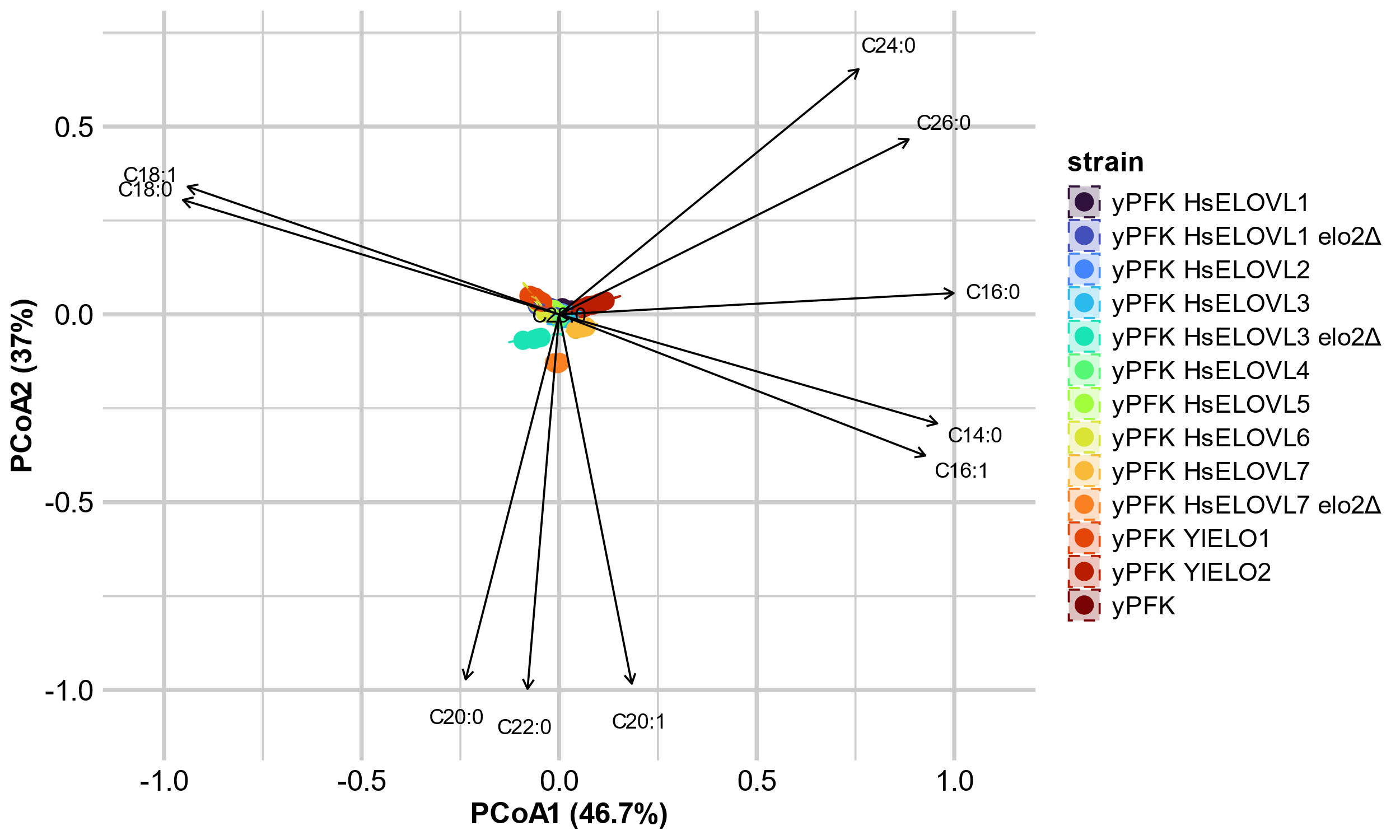
**

**Figure S15 : Ordination of fatty acid profiles of yPFK strains expressing the seven human elongases cultivated without exogenous fatty acid addition and analyzed using the newly developed miniaturized method.** (A) Principal Coordinates Analysis (PCoA) based on Bray-Curtis distances of Hellinger-transformed fatty acid profiles. Points represent individual samples and dashed ellipses indicate 95% confidence intervals for each strain. (B) PCoA with overlaid envfit vectors for individual fatty acids. Arrows indicate the direction and relative contribution of each fatty acid to the observed separation among strains. The separation of points is identical to panel A, but arrows illustrate the variables driving strain differentiation.


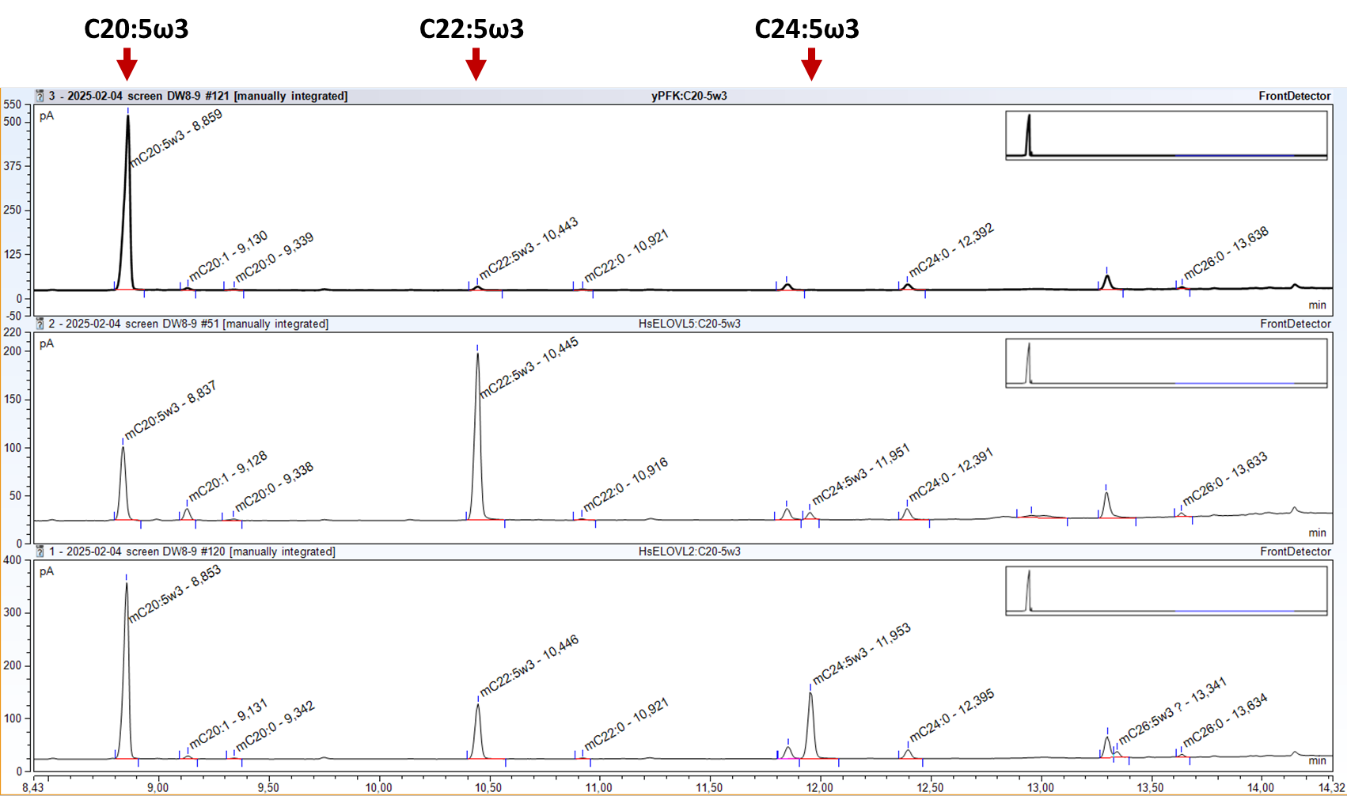


**Figure S16 : HsELOVL2 and HsELOVL5 are able to elongate C20:4ω6 and C20:5ω3 for two elongation cycles.** GC-FID chromatograms of strains yPFK (top panel), yPFK-HsELOVL5 (middle) and yPFK-HsELOVL2 (bottom) cultivated with addition of exogenous C20:5ω3. Elution time for C20:5ω3 and its elongation products are highlighted by red arrows.


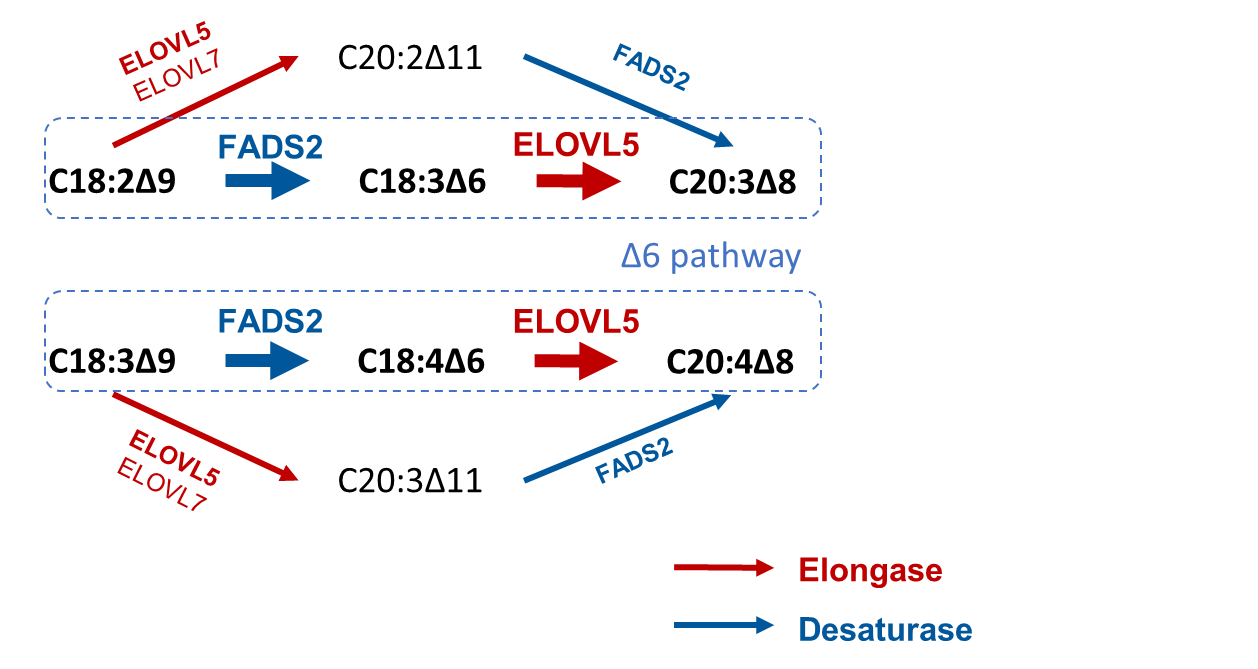


**Figure S17 :** ∆6 elongation pathway (circled in blue dashed line) is preferred over the ∆9 pathway to produce PUFAs in humans. Size of the arrow is proportion to their activity on different substrates and show that FADS2 and ELOVL5 are more active on ∆6 substrates.
